# Reactions of CO_2_ and ethane enable CO bond insertion for production of C3 oxygenates

**DOI:** 10.1038/s41467-020-15849-x

**Published:** 2020-04-20

**Authors:** Zhenhua Xie, Yuanguo Xu, Meng Xie, Xiaobo Chen, Ji Hoon Lee, Eli Stavitski, Shyam Kattel, Jingguang G. Chen

**Affiliations:** 1 Chemistry Division, Brookhaven National Laboratory, Upton, NY 11973 USA; 2 Department of Chemical Engineering, Columbia University, New York, NY 10027 USA; 3 School of Chemistry and Chemical Engineering, Jiangsu University, Zhenjiang, 212013 China; 4 School of Pharmacy, Jiangsu University, Zhenjiang, 212013 China; 5 Department of Mechanical Engineering, State University of New York at Binghamton, NY, 13902 USA; 6 National Synchrotron Light Source-II, Brookhaven National Laboratory, Upton, NY 11973 USA; 7 Department of Physics, Florida A&M University, Tallahassee, FL 32307 USA

**Keywords:** Heterogeneous catalysis, Natural gas, Chemical engineering

## Abstract

Reacting CO_2_ and ethane to synthesize value-added oxygenate molecules represents opportunities to simultaneously reduce CO_2_ emissions and upgrade underutilized ethane in shale gas. Herein, we propose a strategy to produce C3 oxygenates using a tandem reactor. This strategy is achieved with a Fe_3_Ni_1_/CeO_2_ catalyst (first reactor at 600–800 °C) for CO_2_-assisted dehydrogenation and reforming of ethane to produce ethylene, CO, and H_2_, and a RhCo_x_/MCM-41 catalyst (second reactor at 200 °C) enabling CO insertion for the production of C3 oxygenates (propanal and 1-propanol) via the heterogeneous hydroformylation reaction at ambient pressure. In-situ characterization using synchrotron spectroscopies and density functional theory (DFT) calculations reveal the effect of Rh–Co bimetallic formation in facilitating the production of C3 oxygenates. The proposed strategy provides an opportunity for upgrading light alkanes in shale gas by reacting with CO_2_ to produce aldehydes and alcohols.

## Abstract (web summary)

Reacting CO_2_ and ethane to achieve value-added C3 oxygenates offers opportunities to simultaneously reduce CO_2_ emissions and upgrade underutilized ethane in shale gas. Here, the authors report a successful oxygenate production strategy enabled by inserting CO_2_-derived CO into ethane-derived ethylene using a tandem reactor.

## Introduction

In recent decades, increasing anthropogenic CO_2_ emissions^1–3^ and verified huge shale gas reserves^4,5^ have led to studies of effective ways of utilizing these molecules for synthesis of value-added chemicals. The catalytic reduction of CO_2_ by light alkanes (methane, ethane, propane, etc.) has been the subject of many investigations due to its promising role in mitigating CO_2_ emissions and producing chemical intermediates, such as syngas (CO + H_2_)^6–11^, alkenes^9,10,12,13^, and aromatics^13,14^. However, there is a lack of feasible strategy to produce oxygenates directly from CO_2_ and shale gas. Oxygenates (e.g., aldehyde and alcohol) are important chemicals and feedstocks widely used in the automotive, fine chemical, and pharmaceutical industries^15,16^. Thus, it is highly appealing to develop an effective strategy that enables the production of oxygenates by directly inserting CO moiety from CO_2_ into alkenes generated from shale gas.

**Fig. 1:**
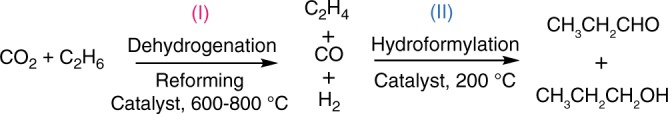
Reaction scheme of the production of C3 oxygenates from the upgrading of CO_2_ and ethane. Ethylene and syngas are produced from the first reactor via the CO_2_-assisted dehydrogenation and reforming of ethane and subsequently used for the hydroformylation reaction in the second reactor to produce propanal and/or 1-propanol.

Traditional hydroformylation, known as an important oxo-synthesis route^17–19^, could be used as an important bridge to achieve the production of oxygenates from CO_2_ and shale gas. It involves the addition of syngas to alkenes to produce aldehydes and/or alcohols with 100% atom efficiency^20^. Syngas can be derived from natural gas, naphtha, or coal by partial oxidation or steam reforming^21,22^. Alkenes are typically produced from the partially oxidative cracking of naphtha or alkanes^23,24^. However, the transportation and storage of the highly toxic syngas and flammable alkenes raise potential safety risks^21,22^. Such drawbacks of the traditional hydroformylation process could be avoided if the feedstocks (alkenes and syngas) produced from the upstream reactor can be directly used for the downstream hydroformylation reaction, i.e., the tandem reactor strategy as shown in Fig. 1.

It has been identified that the catalytic reduction of CO_2_ by ethane (the second most abundant component in shale gas) can proceed with either dry reforming (via C–C bond cleavage) to generate syngas or oxidative dehydrogenation (via C–H bond cleavage) to yield ethylene^11^. Depending on catalyst properties and reaction conditions, the ratio of ethylene/syngas could be tuned^6^, making it possible to use the product mixture as reactants to produce C3 oxygenates (propanal and 1-propanol) from CO_2_ and ethane via the strategy illustrated in Fig. 1.

Compared with the traditional high-pressure homogenous hydroformylation, the proposed strategy would benefit by carrying out reactions at ambient pressure, as illustrated by the thermodynamic analysis of C3 oxygenates at different pressures in Supplementary Fig. 1. This in turn requires efficient heterogeneous catalysts for both reactions to promote the formation of C3 oxygenates. Non-precious FeNi/CeO_2_ catalyst is a good candidate for step (I) due to its role in readily tuning ethylene/syngas ratio using Fe_*x*_/Ni_*y*_ catalysts with different stoichiometries^6^. Different from the ligand complexes used in the traditional homogeneous hydroformylation catalysis, supported metal catalysts should be used for the heterogeneous reaction in step (II). Several previous studies have revealed that Rh, Co, and their bimetallic are the most promising catalysts^21,22,25,26^. However, it is not clear how these catalysts would perform in the tandem configuration with the hydroformylation feeds being directly generated from the reaction of CO_2_ and ethane.

In the present work, we explore the possibility of achieving the strategy to produce C3 oxygenates from CO_2_ and ethane. The experimental conditions are first identified using thermodynamic analyses for promising equilibrium conversions of CO_2_ and ethane into C3 oxygenates. Then, experiments with a tandem reactor validate the feasibility of the proposed reaction strategy using the combination of Fe_3_Ni_1_/CeO_2_ and Rh-based catalysts supported on a high surface area MCM-41 substrate. Finally, in-situ characterizations and density functional theory (DFT) calculations unravel the role of Co species in modifying the structural and electric properties of Rh, enhancing the formation of C3 oxygenates.

## Results

### Thermodynamic analyses of reactions at ambient pressure

**Fig. 2:**
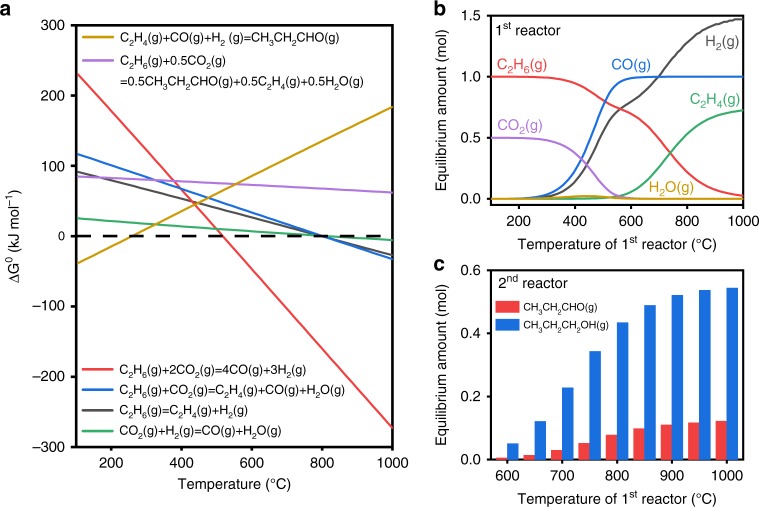
Thermodynamic analyses of the proposed reaction strategy at the atmospheric pressure. **a** Diagram of standard Gibbs free energy change (Δ*G*^0^) of involving reactions along with temperature. **b** Equilibrium species distribution as a function of temperature within the first reactor (feed ratio of C_2_H_6_/CO_2_ = 1 mol/0.5 mol). **c** Equilibrium amount of C3 oxygenates (propanal and 1-propanol) formed at 200 °C within the second reactor using the products of the first reactor, which is maintained within the temperature range of 600–1000 °C. All the thermodynamics were calculated with HSC Chemistry 6.0.

Figure 2a shows that the direct conversion of ethane and CO_2_ into propanal is unfavorable with a highly positive Δ*G*^0^ (purple line). However, it could be achieved using an alternative strategy as shown in Fig. 1 by dividing the overall reaction into two cascade steps: (I) production of ethylene and syngas and (II) subsequent hydroformylation. Figure 2a also shows that both dehydrogenation (blue and black lines) and reforming (red line) of ethane are favorable at high temperatures. In contrast, the hydroformylation of ethylene (golden line) thermodynamically prefers to occur at low temperatures. As a result, there is a thermodynamic gap between steps (I) and (II), which can be bridged using a tandem reactor to run each step within respective favorable temperature regions. To explore the feasibility of this hypothesis, equilibrium analyses were carried out at atmospheric pressure at different temperatures. As illustrated in Fig. 2b, the equilibrium distribution of products strongly depends on the temperature of the first reactor (C_2_H_6_ + CO_2_): syngas is the dominant product below 600 °C, whereas C_2_H_4_ becomes comparable with syngas at above 700 °C. Subsequently, the equilibrium products (mainly CO, H_2_, and C_2_H_4_) from the first reactor obtained at different temperatures are used as the feed for the hydroformylation reaction in the second reactor (200 °C). Figure 2c shows that the second reactor can yield 7–55% of oxygenates (propanal and 1-propanol) as the first reactor (feed ratio of C_2_H_6_/CO_2_ = 1 mol/0.5 mol) is maintained from 600 °C to 1000 °C.

### Catalytic performance tests with single and tandem reactors

**Fig. 3:**
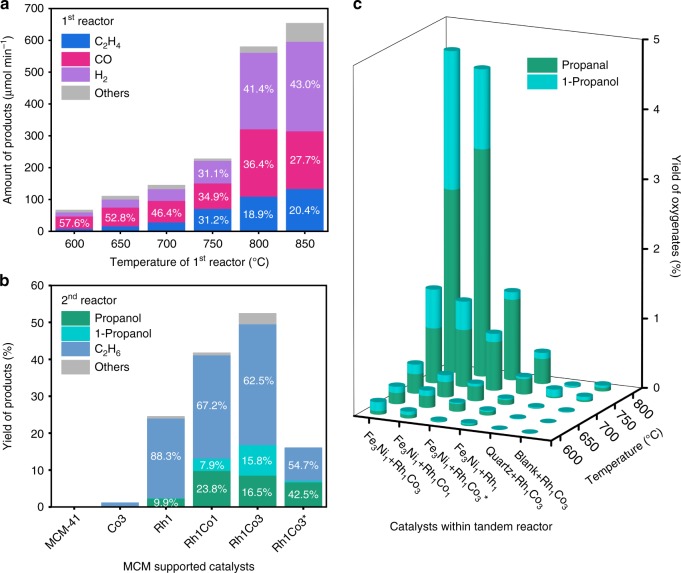
Catalytic performance of different catalysts. **a** Amount of products formed during the reaction of CO_2_ and C_2_H_6_ over Fe_3_Ni_1_/CeO_2_ at different temperatures (600–850 °C). **b** Product yield (C_2_H_4_-based) of the reaction of C_2_H_4_, CO, and H_2_ over the MCM-41 supported Rh-based catalysts at 200 °C. **c** Product yield (C_2_H_6_-based) of the reaction of CO_2_ and C_2_H_6_ within the tandem reactor (first reactor: 600–800 °C, second reactor: 200 °C). Note: the numbers within the bars of Fig. 3a, b indicate product selectivity.

Based on the thermodynamic analyses, flow reactor experiments were performed using a tandem reactor configuration: first reactor (600–850 °C) loaded with Fe_3_Ni_1_/CeO_2_; second reactor (200 °C) loaded with Rh-based catalysts. Figure 3a shows that distributions of C_2_H_4_, CO, and H_2_ from the first reactor strongly depended on the temperature. Below 700 °C, the production of C_2_H_4_ was negligible compared with that of syngas. At 750 °C and above, the C_2_H_4_/CO/H_2_ ratio approached to a typical hydroformylation feed ratio (1 : 1 : 1). As for the second reactor, using pure feed of C_2_H_4_, CO, and H_2_, Fig. 3b and Supplementary Table 2 illustrate that as the Co/Rh bimetallic ratio increased, the yield of oxygenates increased from 2.4% on Rh/MCM-41 to 13.2% and 16.9% on Rh_1_Co_1_/MCM-41 and Rh_1_Co_3_/MCM-41, respectively. Moreover, the selectivity of 1-propanol also increased and even became comparable with propanal (15.8% vs. 16.5%) on Rh_1_Co_3_/MCM-41, offering a way to control the alcohol/aldehyde ratio. In addition, the oxygenate selectivity could also be affected by temperature that 42.5% of propanal was obtained on Rh_1_Co_3_/MCM-41 at 180 °C (denoted as Rh_1_Co_3_* in Fig. 3b). As indicated by the Supplementary Table 2 and Supplementary Figs. 3–6, the measured propanal/1-propanol ratios in the second reactor were above 1.0, higher than the equilibrium ratio of 0.26 at 200 °C (Supplementary Fig. 15), indicating that the interconversion between propanal and 1-propanol did not reach equilibrium. For the tandem reactor configuration in Fig. 3c, C3 oxygenates were detected on the Rh_1_Co_*x*_/MCM-41 catalysts as the first reactor ran at above 600 °C. Control experiments with MCM-41, quartz sand, and blank tubing exhibited negligible oxygenates production (Supplementary Table 2–4). The highest yield of C3 oxygenates (~4.7%) was achieved by using the Fe_3_Ni_1_/CeO_2_ (at 800 °C) and Rh_1_Co_3_/MCM-41 (at 200 °C) catalysts. As shown in Supplementary Fig. 7, a further increase in the C3 oxygenate yield toward 7.2% could be achieved by increasing the catalyst loading and modifying the reaction conditions. In addition, the durability of the Fe_3_Ni_1_/CeO_2_ catalyst, within the testing period of 31 h (Supplementary Fig. 8), indicated a stable feed supply for the downstream reactor. These results validate the feasibility of using the tandem reactor configuration to convert CO_2_ and ethane into C3 oxygenates.

It is noted that the values for conversion, turnover frequency (TOF), and oxygenates yield (Supplementary Figs. 2–6 and Supplementary Table 2) over Rh_1_Co_3_/MCM-41 were higher than the sum of that of Rh/MCM-41 and Co_3_/MCM-41, indicating a synergistic effect between Rh and Co in the bimetallic catalysts. To elucidate the origin of such effect, the metal particle size distributions were obtained based on the high-angle annular dark-field (HAADF) images shown in Supplementary Figs. 9–11. Elemental mappings in Supplementary Figs. 10 and 11 indicated a uniform distribution of Co around Rh particles. As the Co/Rh ratio increased from 0 : 1 to 1 : 1 and 3 : 1, metal particle size decreased from ~2.3 nm to ~1.7 nm and ~1.4 nm with a narrower distribution. The CO uptake values also increased from 57.6 μmol g^−1^ on Rh/MCM-41 to 78.9 and 108.1 μmol g^−1^ on Rh_1_Co_1_/MCM-41 and Rh_1_Co_3_/MCM-41, respectively. Thus, the presence of Co promoted the dispersion of the Rh particles.

### In-situ X-ray absorption fine structure analysis

**Fig. 4:**
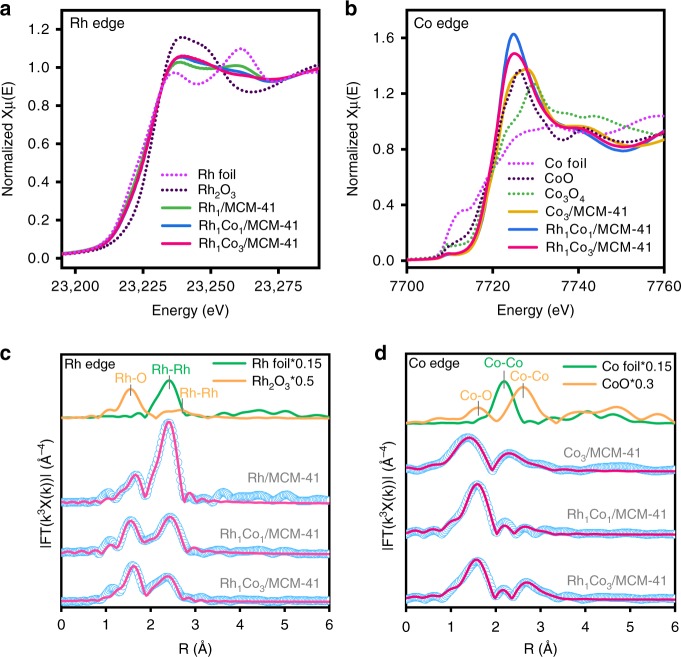
Structural analyses of mono- and bimetallic MCM-supported Rh-based catalysts. **a**, **b** XANES spectra of Rh and Co K-edges. **c**, **d** Fourier transformed EXAFS spectra (sky blue circles) and fittings (deep pink line) of Rh and Co K-edges.

In-situ X-ray absorption near edge structure (XANES) and extended X-ray absorption fine structure (EXAFS) spectra of Rh K-edge and Co K-edge were measured to explore the electronic properties and local environments under hydroformylation conditions. Referring to the XANES spectra of metal and oxides standards in Fig. 4a, Rh in Rh/MCM-41 was slightly oxidized and it became more oxidized with the presence of Co. As shown in Fig. 4c, the EXAFS signals in R space indeed showed a significant peak located between 1.5–2.0 Å, correlating with the coordination by low-Z elements (e.g., C or O). It is difficult to distinguish the contributions from Rh–C or Rh–O bonds due to their similar bond length. The possibility of Rh–O bond from bulk Rh oxides could be ruled out due to the lack of corresponding Rh–Rh peaks from bulk oxides. Nevertheless, given the fine assembles from HAADF images, a Rh–O contribution from the MCM-41 substrate could not be excluded. Temperature-programmed reduction (TPR) profiles on both the mono- and bimetallic Rh-based catalysts show that besides the reduction of bulk Rh oxides at 100–110 °C, a peak located within 275–285 °C was observed. The latter should correlate with the reduction of the Rh species interacted with the silica framework (Supplementary Fig. 12). Rh–C interaction, especially from CO adsorption, could also be possible due to the formation of carbonyl on the Rh surface^27,28^. Thus, the Rh-low Z peak was assigned to both of Rh–C and Rh–O contributions. The peak located between 2.0 Å and 3.0 Å was mainly associated with the Rh-M (Rh or Co) bonds. With increasing Co/Rh ratio from 0 : 1 to 1 : 1 and 3 : 1, the Rh-M peak noticeably decreased, validating the reduction of Rh particle size as suggested by the transmission electron microscopy (TEM) and CO chemisorption experiments. In Fig. 4b, the Co K-edge spectrum of Co_3_/MCM-41 showed a strong white line due to the strong interaction between Co^2+^ and silica framework^29^. As shown in Fig. 4d, nearly negligible Co–Co bond from bulk oxides was observed on the bimetallic samples, suggesting that the majority of the Co species was highly dispersed on the MCM-41 support. However, it is noted that the bimetallic samples showed an even stronger white line, indicating a decrease of electron density at the Fermi level that should result in weak or negligible CO adsorption. Therefore, the majority of Co species that strongly interacted with the silica framework^29^ was probably not involved in catalysis. However, given the HAADF and CO chemisorption results, the interaction of CoO_*x*_ species with Rh was most likely responsible for the increase of Rh dispersion. Rh_1_Co_1_/MCM-41 and Rh_1_Co_3_/MCM-41 exhibited much higher formation rate of C3 oxygenates (1.12 min^−1^ and 1.04 min^−1^, respectively) than Rh/MCM-41 (0.26 min^−1^) by a factor of 4–5 times. Such increase cannot be explained solely by the increase in Rh dispersion (1.4–1.9 times). Moreover, the 1-propanol selectivity also increased with the Co/Rh ratio. It is noted that the EXAFS fitting results of both Rh and Co edges (Supplementary Table 10) indicated the presence of Rh–Co bond on the bimetallic samples. Thus, the increase of the total C3 formation rate and 1-propanol selectivity with the Co/Rh ratio most likely resulted from the fraction of Co that formed bimetallic bond with Rh. To validate this hypothesis, DFT calculations were performed on the Rh and RhCo surfaces as follows.

### Density functional theory calculations

**Fig. 5:**
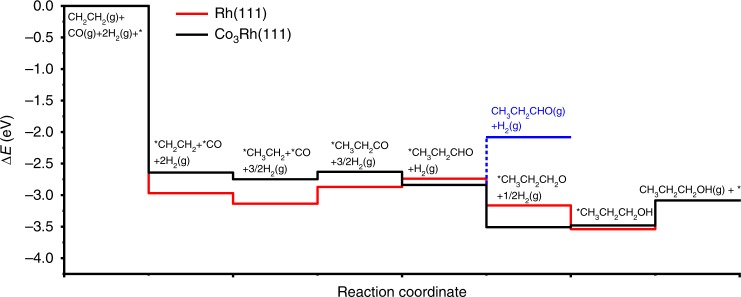
DFT calculations of reaction pathways on Rh(111) and Co_3_Rh(111). DFT calculated energy change (∆*E*) for hydroformylation into propanal and further hydrogenation into 1-propanol on monometallic Rh and bimetallic RhCo surfaces.

The enthalpy change for ethylene hydroformylation to propanal and 1-propanol along the reaction pathways shown in Fig. 5 were calculated on Rh(111) and bulk-terminated-Co_3_Rh(111) [Co_3_Rh(111) hereafter] surfaces. The binding energies of involved reaction intermediates at their optimized configurations (Supplementary Fig. 13) were calculated (Supplementary Table 11) to compute the enthalpy change (Δ*E*) for the formation of propanal and 1-propanol on the two surfaces. The DFT calculated Δ*E* values along the reaction pathways as illustrated in Fig. 5 showed that the presence of Co on the RhCo bimetallic surface facilitated the formation of 1-propanol due to stronger binding of oxygenate intermediates such as *CH_3_CH_2_CHO and *CH_3_CH_2_CH_2_O compared with Rh(111). Thus, a RhCo bimetallic catalyst with abundant surface Co sites should be expected to show higher selectivity toward 1-propanol formation in the hydroformylation reaction of C_2_H_4_.

Further DFT calculations were performed to determine the reaction energy (Δ*E*) and activation energy (*E*_a_) of selected steps for the formation of propanal and 1-propanol. The DFT calculated Δ*E* and *E*_a_ for the first hydrogenation step to form *CH_3_CH_2_ were (0.33 and 0.77 eV) and (0.42 and 0.68 eV) on Rh(111) and Co_3_Rh(111), respectively. The Δ*E* and *E*_a_ values for the second hydrogenation reaction to form propanal (*CH_3_CH_2_CHO) after the formation of *CH_3_CH_2_CO via inserting *CO into *CH_3_CH_2_ were (0.59 and 0.82 eV) and (0.31 and 0.46 eV) on Rh(111) and Co_3_Rh(111), respectively. The hydrogenation of propanal (*CH_3_CH_2_CHO + *H → *CH_3_CH_2_CH_2_O + *) had Δ*E* and *E*_a_ values of (0.04 and 0.55 eV) and (−0.29 and 0.41 eV) on Rh(111) and Co_3_Rh(111), respectively. It was noted that the calculated *E*_a_ values for the hydrogenation reactions along reaction pathways were lower on Co_3_Rh(111) compared with Rh(111). Thus, the DFT results suggested that hydrogenation of the hydroformylation intermediates was promoted due to the presence of Co on the bimetallic surface. On Rh(111), the desorption of *CH_3_CH_2_CHO was uphill by 0.66 eV (blue line) and its hydrogenation to *CH_3_CH_2_CH_2_O had an *E*_a_ of 0.55 eV, thus competing with each other. However, the entropy contribution made desorption more favorable leading to the formation of *CH_3_CH_2_CHO as the major product on Rh(111). In contrast, on Co_3_Rh(111), *CH_3_CH_2_CHO desorption was uphill by 0.76 eV and its hydrogenation to *CH_3_CH_2_CH_2_O had an *E*_a_ of 0.41 eV. This showed that the formation of *CH_3_CH_2_CH_2_O, a critical intermediate for 1-propanol formation, was more favorable on Co_3_Rh(111) compared to Rh(111) due to stronger bindings of *CH_3_CH_2_CHO and *CH_3_CH_2_CH_2_O on Co_3_Rh(111). The hydrogenation of *CH_3_CH_2_CH_2_O to *CH_3_CH_2_CH_2_OH had Δ*E* of 0.63 eV and a relatively high *E*_a_ of 1.42 eV on Co_3_Rh(111), indicating that this step was potentially rate-limiting to form 1-propanol. Overall, in agreement with the experimental results, DFT calculations predicted an enhanced production of C3 oxygenates, especially 1-propanol on the Rh–Co bimetallic catalysts.

## Discussion

In summary, we have proposed and demonstrated a successful oxygenate production strategy enabled by inserting CO_2_-derived CO into ethane-derived ethylene using a tandem reactor (with Fe_3_Ni_1_/CeO_2_ and RhCo_x_/MCM-41 catalysts) at ambient pressure. The presence of Co species could not only promote oxo-synthesis activity by increasing Rh dispersion, but also promote C3 oxygenates formation especially 1-propanol due to favorable hydrogenation of hydroformylation intermediates on the Rh–Co bimetallic surface. This study offers a promising way to simultaneously convert CO_2_ and light alkanes in shale gas into value-added aldehydes and alcohols.

## Methods

### Preparation of Rh-based mono- and bimetallic catalysts

All regents were used without purification. Unless otherwise noted, all of the chemicals (Fe(NO_3_)_3_ ∙ 9H_2_O, Ni(NO_3_)_2_ ∙ 6H_2_O, Co(NO_3_)_2_ ∙ 6H_2_O, Rh(NO_3_)_3_ ∙ *x*H_2_O, CeO_2_, and MCM-41) were purchased from Sigma Aldrich. The information of precursors, loading amount and atomic ratio were listed in Supplementary Table 1. All the catalysts were synthesized using an incipient wetness co-impregnation method over the CeO_2_ or MCM-41 supports with an aqueous solution of the respective metal precursors. All the catalysts were dried at 80 °C overnight and then calcined at 290 °C for 2 h with a heating ramp rate of 0.8 °C min^−1^.

### Characterization of catalysts

Pulse CO chemisorption was performed in an AMI-300ip (Altamira) instrument. As-prepared catalyst (~100 mg) was pretreated under He atmosphere (50 ml min^−1^) at 120 °C for 30 min and then cooled to 30 °C. Then, the sample was heated to 200 °C (10 °C min^−1^) and held for 1 h in a mixture of 10% H_2_ in Ar (totally 50 ml min^−1^), and cooled down in He (50 ml min^−1^) for degassing before pulsing 10% CO in He (590 μL loop). The amount of chemisorbed CO was used to estimate the number of metal active sites on the catalyst with an assumption of CO/metal ratio being 1 : 1.

The same AMI-300ip (Altamira) instrument was used for TPR measurements. For TPR tests, as-prepared sample (~100 mg) was pretreated under He atmosphere (50 ml min^−1^) at 120 °C for 30 min and then cooled to 40 °C. TPR test was subsequently performed in a mixture of 10% H_2_ in Ar (total 50 ml min^−1^) with a heating rate of 10 °C min^−^^1^ to 450 °C. A thermal conductivity detector (TCD) was used to record the hydrogen consumption profile as a function of reduction temperature, allowing a quantitative comparison of the reducibility of active metals in different catalysts.

Metal particle distribution on the spent Rh-based catalysts was characterized using HAADF imaging within an FEI Talos F200X TEM (operated at 200 keV). Scanning TEM–energy dispersive spectroscopy mapping was performed using the same instrument. Spent samples were ultrasonically dispersed in ethanol for 10 min. Afterwards, a droplet was dripped onto lacey carbon film supported on copper grid and fully dried before use.

The in-situ XAFS experiments were carried out at beamline 8-ID (ISS) of the National Synchrotron Light Source II at Brookhaven National Laboratory. The in-situ Rh K-edge (23,220 eV) and Co K-edge (7709 eV) XAFS spectra were collected for MCM-41 supported catalysts, as well as metal foils (Rh and Co) and metal oxide standards (Rh_2_O_3_, Co_3_O_4_, and CoO). Approximately 100 mg of sample (60 − 80 mesh) was loaded into a home-designed in-situ copper micro-channel reactor^6^. Graphite carbon paper was used as the reactor window material for transmission and fluorescence modes. Quartz wool was packed at both sides of the sample to fix the catalyst bed. The sample was exposed to the reaction stream (C_2_H_4_/CO/H_2_/He = 3/3/3/3 ml min^−1^) at 200 °C for 60 min. To reduce thermal disordering contribution, the catalyst bed was cooled to room temperature under the reaction stream. About 30 scans were collected and merged as an individual spectrum to improve the signal-to-noise ratio. Between two scans, an interval time of 45 s was imposed for rest to reduce sample damage potentially induced by the high-flux (10^14^ @ 10 keV) X-ray beam exposure. Data processing was preformed using the IFEFFIT package. Rh and Co foils were used as standard references to calibrate energy shift and obtain passive electron reduction factor (S_0_^2^). It should be noted that the contributions from both metal and nonmetal (low-Z elements) neighbors were significant in the Rh spectra, which complicated the EXAFS analysis. Therefore, a similar method was adopted from ref. ^30^, with which the residuals of the fits to the metal contribution were separately fit to approximate the low-Z contribution. In addition, owing to the similarity of Rh–C and Rh–O EXAFS signals in the first nearest shell, they were treated together as the Rh-low Z interactions. Based on ref. ^30^, the Rh–Rh(Co) and Rh-low *Z* interactions were analyzed with the phase shifts and back-scattering amplitudes obtained from the Feff calculations of Rh foil and Rh_2_O_3_ structures, respectively. Moreover, for the Rh_1_Co_3_ (Rh_1_Co_1_) bimetallic samples, the EXAFS spectra of both Rh K-edge and Co K-edge were fitted simultaneously with additional constraints^30^ of coordination number (CN) relationship, bond length (R) and structure disordering (*σ*^2^), i.e., CN_Rh-Co_/CN_Co-Rh_ = 3 : 1 (or 1 : 1), R_Rh-Co_ = R_Co-Rh_, *σ*^2^_Rh-Co_ = *σ*^2^_Co-Rh_. For Co edge, the EXAFS signals were fitted based on the information of Co foil and CoO structures.

### Catalytic performance evaluation

Tandem reactor experiments were carried out within two quartz tubes (7 mm ID, 9.6 mm OD) heated separately by two tandem furnaces (Thermo Scientific Lindberg/Blue M) under the atmospheric pressure. For a typical experiment, ~300 mg (Fe_3_Ni_1_/CeO_2_) and ~200 mg (Rh_1_Co_*x*_/MCM-41, *x* = 0, 1, or 3) of catalysts (60–80 mesh) were loaded into the first and second flow reactors, respectively. Approximately 100 mg of acid-purified quartz particle (60–80 mesh) was used for the dilution of each catalyst bed to reduce heat transport limitations. In prior to each reaction, the catalyst in the first reactor was pretreated under a mixture of H_2_ and Ar (5/5 ml min^−1^) at 600 °C for 1 h, during which process the catalyst in the second reactor was bypassed and maintained under Ar atmosphere. After the reduction, the catalyst bed in each reactor was heated to desired temperatures, and subsequently exposed to the reaction stream of C_2_H_6_/CO_2_/Ar (6/3/3 ml min^−1^) at the atmospheric pressure. The outlet of the first reactor was connected immediately to the inlet of the second reactor to ensure all the effluents enter into the second reactor. In addition, the outlet gasline of the second reactor was wrapped with heating tapes at 150 °C to avoid any condensations of water vapor and oxygenates. The exhaust was analyzed by an Agilent 7890B gas chromatography (PLOT Q and MOLESEIVE columns) equipped with a thermal conductivity detector and a flame ionized detector. Single reactor experiments followed the similar procedure. Water vapor was calibrated based on oxygen balance by running the reverse water-gas shift reaction with a PtCo/CeO_2_ catalyst at different temperatures. The element balances of carbon, hydrogen, and oxygen were 100 ± 2%, 100 ± 3, and 100 ± 3%, respectively. Conversion (*X*), TOF, selectivity (*S*), and yield (*Y*) at steady state were calculated as follows:

1 $$X_i = \frac{{[F_i]_{{\mathrm{in}}} - [F_i]_{{\mathrm{out}}}}}{{[F_i]_{{\mathrm{out}}}}} \times 100\%$$

2 $${\mathrm{TOF}}_i = \left| {\frac{{[F_i]_{{\mathrm{in}}} - [F_i]_{{\mathrm{out}}}}}{{{\mathrm{CO}}\,{\mathrm{uptake}} \cdot m_{{\mathrm{catalyst}}}}}} \right|$$

where [*F*_*i*_]_in_ and [*F*_*i*_]_out_ referred to the inlet and outlet molar flow rate, respectively, of reactant *i* corrected by the Ar molar flow rate (mol min^−1^); CO uptake (μmol g^−1^) and *m*_catalyst_ (mg) indicated the number of active site per unit mass of catalyst and the mass of catalyst, respectively.

The selectivity (*S*) of carbon containing species was defined separately for the first reactor, second reactor, and tandem reactor. For the first reactor (reaction of CO_2_ and C_2_H_6_, C_2_H_6_-based selectivity),

3 $$S_i = \frac{{[F_i]_{{\mathrm{out}}}}}{{[F_{{\mathrm{C}}_{\mathrm{2}}{\mathrm{H}}_{\mathrm{6}}}]_{{\mathrm{in}}} - [F_{C_2H_6}]_{{\mathrm{out}}}}} \times \frac{{n_{{\mathrm{carbon - atoms,}}i}}}{{n_{{\mathrm{carbon - atoms,C}}_{\mathrm{2}}H_6}}} \times 100\%$$

where the species *i* could be CO, CH_4_, C_2_H_4_, C_3_H_6_, C_3_H_8_, C_4_H_8_, C_4_H_10_; *n* is the number of carbon from C_2_H_6_ in species *i*. It should be noted that the CO reported here is the part produced from C_2_H_6_ via dry reforming reaction. Based on the oxygen balance, *S*_CO_ was calculated in the following:

4 $$S_{\mathrm{CO}} = \frac{{\left( {[F_{{\mathrm{CO}}}]_{{\mathrm{out}}}} \right) - [F_{{\mathrm{H}}_{\mathrm{2}}{\mathrm{O}}}]_{{\mathrm{out}}}/2}}{{[F_{{\mathrm{C}}_{\mathrm{2}}{\mathrm{H}}_{\mathrm{6}}}]_{{\mathrm{in}}} - [F_{C_2H_6}]_{{\mathrm{out}}}}} \times \frac{{n_{{\mathrm{carbon - atoms,CO}}}}}{{n_{{\mathrm{carbon - atoms,C}}_{\mathrm{2}}{\mathrm{H}}_6}}} \times 100\%$$

For the second reactor (reaction of C_2_H_4_, CO, and H_2_, C_2_H_4_-based selectivity),

5 $$S_i = \frac{{[F_i]_{{\mathrm{out}}}}}{{[F_{C_{\mathrm{2}}H_{\mathrm{4}}}]_{{\mathrm{in}}} - [F_{C_2H_6}]_{{\mathrm{out}}}}} \times \frac{{n_{{\mathrm{carbon - atoms,}}i}}}{{n_{{\mathrm{carbon - atoms,C}}_{\mathrm{2}}{\mathrm{H}}_4}}} \times 100\%$$

where the species *i* could be C_2_H_6_, C_3_H_6_, C_3_H_8_, C_4_H_8_, C_4_H_10_, C_3_H_6_O (propanal) and C_3_H_8_O (1-propanol); *n* is the number of carbon from C_2_H_4_ in species *i*. Note: *n* should be 2 for C_3_H_6_O and C_3_H_8_O.

For the tandem reactor, it follows the same definition as that of the first reactor with a C_2_H_6_-based selectivity. Species *i* could be CO, CH_4_, C_2_H_4_, C_3_H_6_, C_3_H_8_, C_4_H_8_, C_4_H_10_, C_3_H_6_O (propanal) and C_3_H_8_O (1-propanol). Note: *n* was chosen as 3 for C_3_H_6_O and C_3_H_8_O.

The yield (*Y*) of carbon containing species was the product of conversion and selectivity, i.e.,

6 $$Y_i = X_i \cdot S_i$$

### Density functional theory calculation methods

Spin polarized periodic DFT^31,32^ calculations were performed using Vienna Ab-Initio Simulation Package (VASP) code^33,34^. Projector augmented wave potentials were used to describe the core electrons with the generalized gradient approximation^35,36^, using PW91 functionals^37^. The Kohn–Sham one-electron wave functions were expanded by using a plane wave basis set with a kinetic energy cutoff of 400 eV. The Brillouin zone was sampled using a 3 × 3 × 1 *k*-point grid in the Monkhorst–Pack scheme^38^.

The Rh(111) surface was modeled using a four layer 4 × 4 surface slab. The bulk-terminated Co_3_Rh(111) surface was cleaved using a L_12_-Co_3_Rh cubic crystal structure. A 15 Å thick vacuum was added along the direction perpendicular to the surface in the initial slab model to avoid the artificial interactions between the slab and its periodic images. During geometry optimization, the atoms in the top two layers were allowed to relax while the atoms in the bottom two layers were fixed. Ionic positions were optimized until Hellman-Feynman force on each ion was smaller than 0.02 eV/Å.

The binding energy (BE) of an adsorbate is calculated as:

7 $${\mathrm{{BE}}}_{\mathrm{{adsorbate}}} = {\mathrm{{E}}}_{\mathrm{{slab + adsorbate}}} - {\mathrm{{E}}}_{\mathrm{{slab}}} - {\mathrm{{E}}}_{\mathrm{{adsorbate}}}$$

where E_slab+adsorbate_, E_slab_, and E_adsorbate_ are the total energies of slab with adsorbate, clean slab, and adsorbate species in gas phase, respectively.

The transition state of a chemical reaction was located using the climbing image nudged elastic band method implemented in VASP^39^. The activation energy (*E*_a_) of a chemical reaction is defined as the energy difference between the initial and transition states.

## Supplementary information

Supplementary Information

## Data Availability

The data that support the findings of this study are available from the corresponding authors upon reasonable request.
