# Supplementary material for: Reactions of CO2 and ethane enable CO bond insertion for production of C3 oxygenates
Source: Nat Commun. 2020 Apr 20;11:1887. doi: 10.1038/s41467-020-15849-x (PMC7170877; doi:10.1038/s41467-020-15849-x)
Supplement: Supplementary file 1 — Supplementary Information [file 41467_2020_15849_MOESM1_ESM.pdf]

Supplementary Information

Reactions of CO_2 and Ethane Enable CO Bond Insertion for Production of C3 Oxygenates

Xie et al.

Table of Contents

Supplementary Figures

Supplementary Figure 1. Thermodynamic analyses of the pressure effect on the formation of C3 oxygenates from the tandem reactor....3
Supplementary Figure 2. Catalytic performance of the MCM-41 supported catalysts....4
Supplementary Figure 3. Catalytic performance of the Rh/MCM-41 catalyst....5
Supplementary Figure 4. Catalytic performance of the Rh_1Co_1/MCM-41 catalyst....6
Supplementary Figure 5. Catalytic performance of the Rh_1Co_3/MCM-41 catalyst....7
Supplementary Figure 6. Catalytic performance of the Rh_1Co_3*/MCM-41 catalyst....8
Supplementary Figure 7. Long-term evaluation of the catalytic performance of the tandem reactor configuration....9
Supplementary Figure 8. Stability test of the spent Fe_3Ni_1/CeO_2 catalyst at 750 ℃....10
Supplementary Figure 9. Electron microscopy imaging of the spent Rh/MCM-41 catalyst after exposed to hydroformylation reaction stream for 10 h....11
Supplementary Figure 10. Electron microscopy imaging of the spent Rh_1Co_1/MCM-41 catalyst after exposed to hydroformylation reaction stream for 10 h....12
Supplementary Figure 11. Electron microscopy imaging of the spent Rh_1Co_3/MCM-41 catalyst after exposed to hydroformylation reaction stream for 10 h....13
Supplementary Figure 12. Temperature-programmed H_2-reduction profiles of the fresh MCM-41 supported catalysts....14
Supplementary Figure 13. DFT optimized geometries of intermediate species on Rh(111)....15
Supplementary Figure 14. Thermodynamic analysis of hydroformylation and hydrogenation reactions of ethylene as a function of temperature....16
Supplementary Figure 15. Thermodynamic calculations for the hydrogenation of propanal to 1-propanol....17
Supplementary Figure 16. Effect of water vapor and residence time on the catalytic performance of the Rh_1Co_3/MCM-41 catalyst....18

Supplementary Tables

Supplementary Table 1 Sample information (metal loading amount, atomic ratio and CO uptake values) of different catalysts....19
Supplementary Table 2 Catalytic performance of MCM-41 supported catalysts within the 2nd reactor at 200 ℃....20
Supplementary Table 3 Catalytic performance of blank tube+Rh_1Co_3/MCM-41 within tandem reactors....21
Supplementary Table 4 Catalytic performance of quartz+Rh_1Co_3/MCM-41 within tandem reactors....22
Supplementary Table 5 Catalytic performance of Fe_3Ni_1/CeO_2 within the 1st reactor....23
Supplementary Table 6 Catalytic performance of Fe_3Ni_1/CeO_2+Rh/MCM-41 within tandem reactors....24
Supplementary Table 7 Catalytic performance of Fe_3Ni_1/CeO_2+Rh_1Co_1/MCM-41 within tandem reactors....25

Supplementary Table 8 Catalytic performance of Fe_3Ni_1/CeO_2+Rh_1Co_3/MCM-41 within tandem reactors....26
Supplementary Table 9 Catalytic performance of Fe_3Ni_1/CeO_2+Rh_1Co_3*/MCM-41 within tandem reactors....27
Supplementary Table 10 EXAFS fitting results of Rh K-edge and Co K-edge for mono- and bimetallic Rh catalysts under the hydroformylation reaction stream....28
Supplementary Table 11 DFT calculated binding energies of intermediates on Rh(111) and Co_3Rh(111) surfaces....29

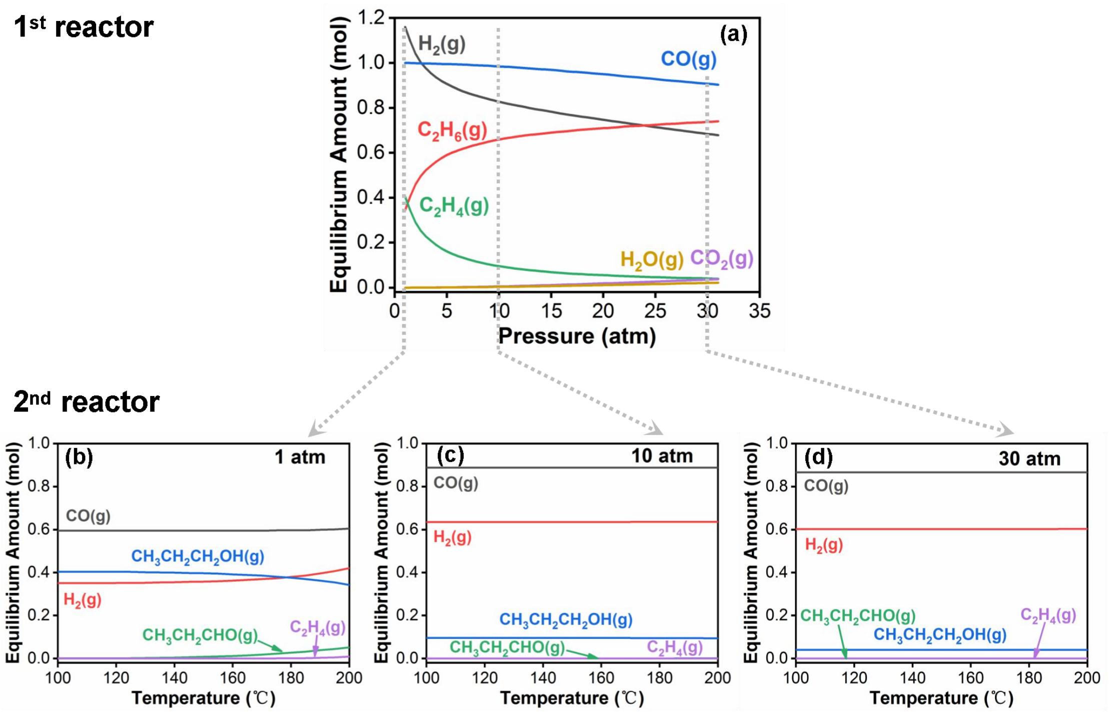

Supplementary Figure 1. Thermodynamic analyses of the pressure effect on the formation of C3 oxygenates from the tandem reactor. **(a)** Equilibrium species distribution as a function of pressure of the 1st reactor (750 ℃, feed ratio of C_2H_6/CO_2=1 mol/0.5 mol). **(b)**, **(c)**, **(d)** Equilibrium species distribution as a function of temperature of the 2nd reactor using the products of the 1st reactor which is maintained at 750 ℃ and 1 atm, 10 atm and 30 atm, respectively. All the thermodynamics were calculated with HSC Chemistry 6.0. Note: the thermodynamics indicate that the higher the pressure within the 1st and 2nd reactor the lower the equilibrium amount of the total C3 oxygenates (propanal and 1-propanol).

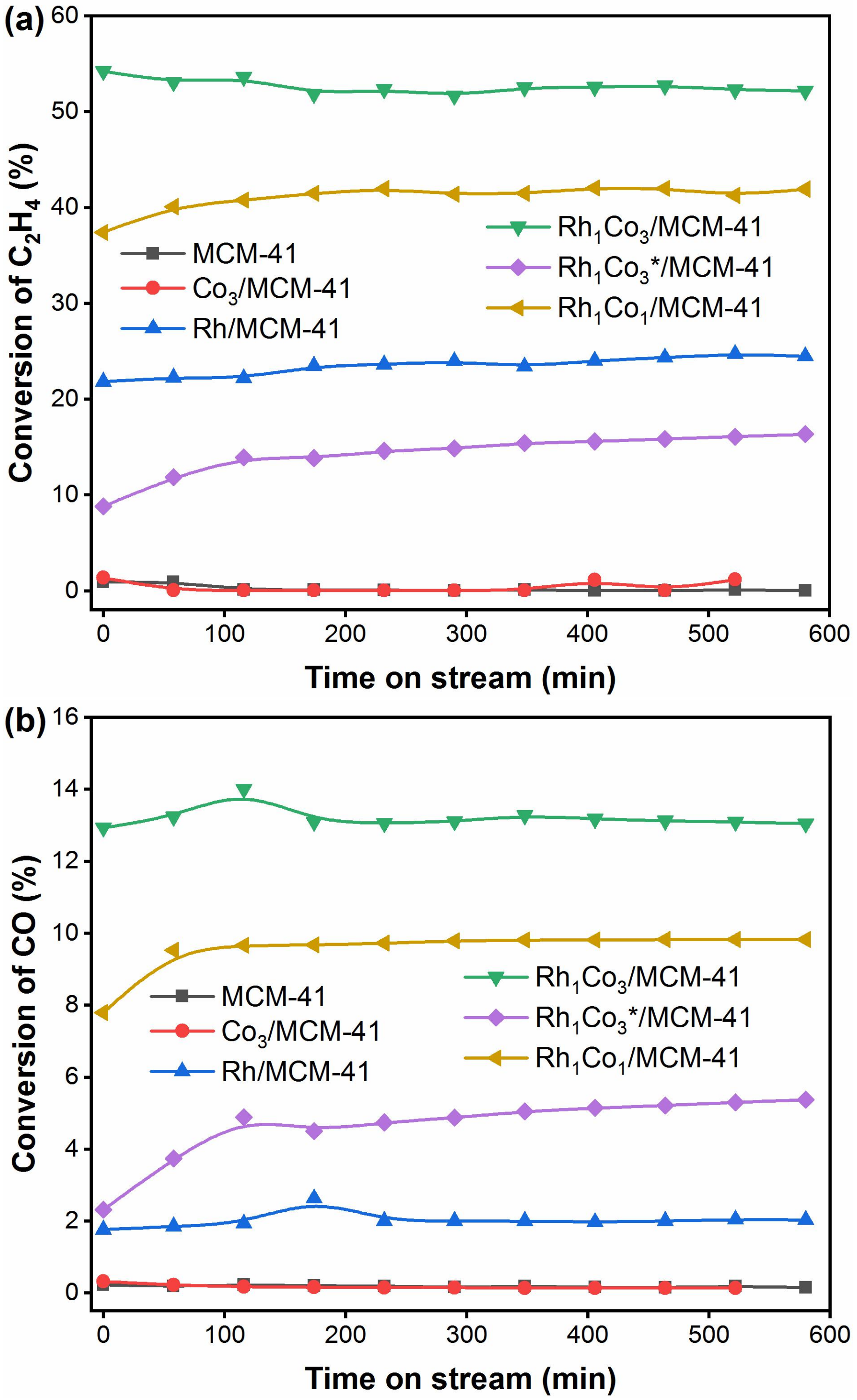

Supplementary Figure 2. Catalytic performance of the MCM-41 supported catalysts. **(a)**, **(b)** Conversion of C_2H_4 and CO on MCM-41, Co_3/MCM-41, Rh/MCM-41, Rh_1Co_1/MCM-41, and Rh_1Co_3/MCM-41 at 200 ℃ and on Rh_1Co_3*/MCM-41 at 180 ℃. Reaction conditions: C_2H_4/CO/H_2/Ar=3/3/3/3 ml/min, catalyst mass=200 mg, 60-80 mesh, diluted with 100 mg of acid-purified quartz particles (60-80 mesh), atmospheric pressure.

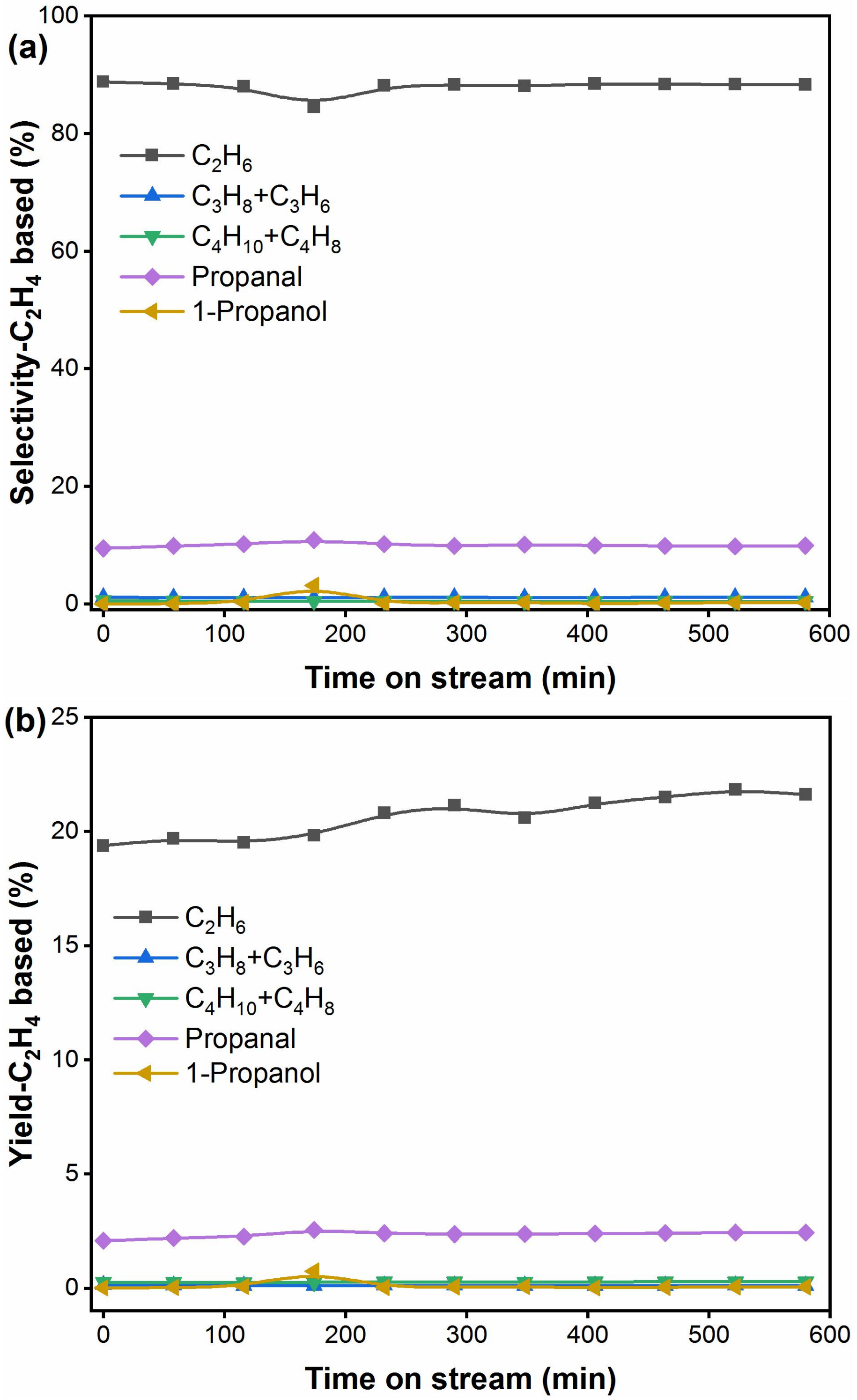

Supplementary Figure 3. Catalytic performance of the Rh/MCM-41 catalyst. **(a)**, **(b)** Selectivity and yield of the carbon containing products. Reaction conditions: 200 ℃, $C_2H_4/CO/H_2/Ar$=3/3/3/3 ml/min, catalyst mass=200 mg, 60-80 mesh, diluted with 100 mg of acid-purified quartz particles (60-80 mesh), atmospheric pressure.

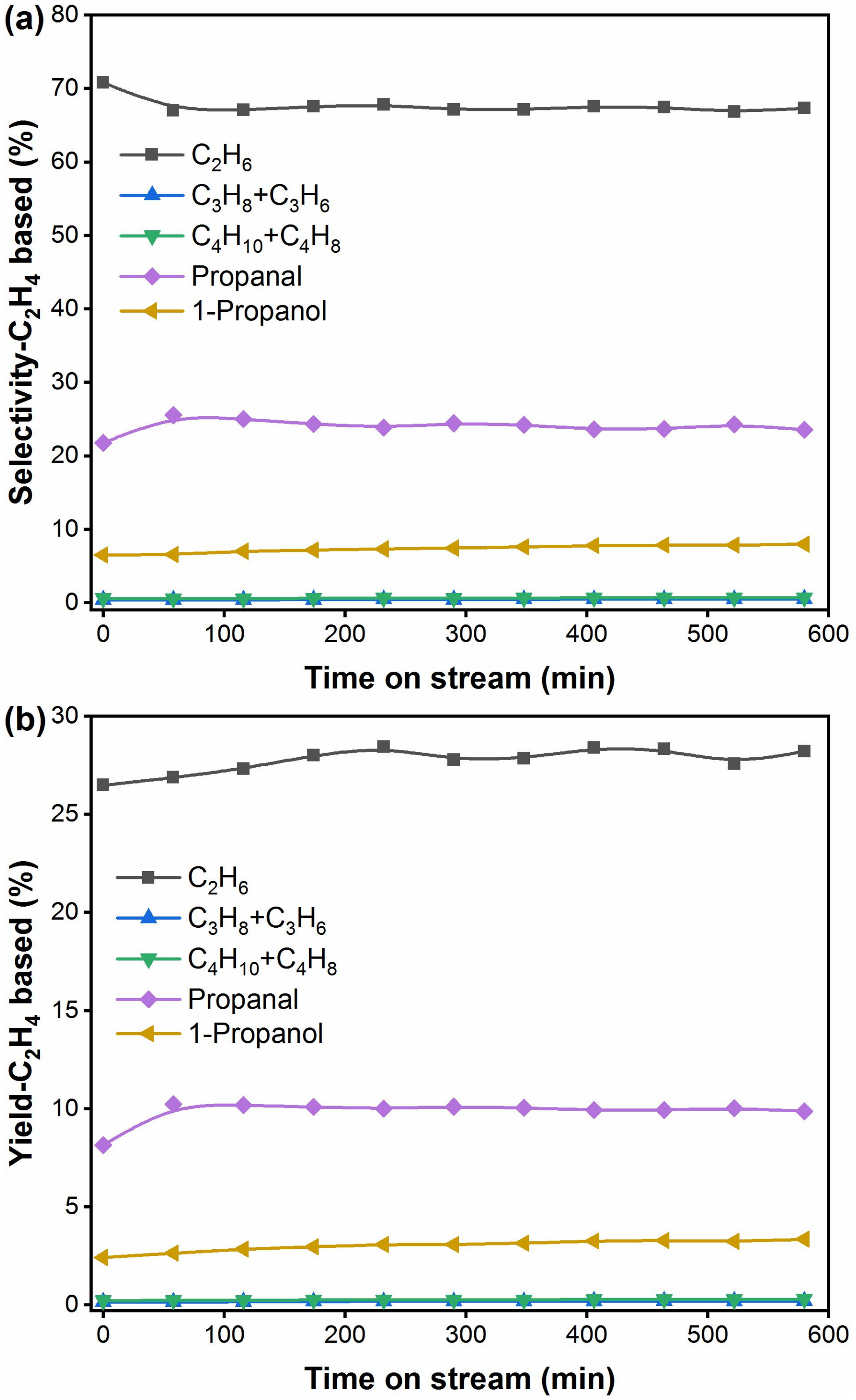

Supplementary Figure 4. Catalytic performance of the Rh_1Co_1/MCM-41 catalyst. **(a)**, **(b)** Selectivity and yield of the carbon containing products. Reaction conditions: 200 ℃, C_2H_4/CO/H_2/Ar=3/3/3/3 ml/min, catalyst mass=200 mg, 60-80 mesh, diluted with 100 mg of acid-purified quartz particles (60-80 mesh), atmospheric pressure.

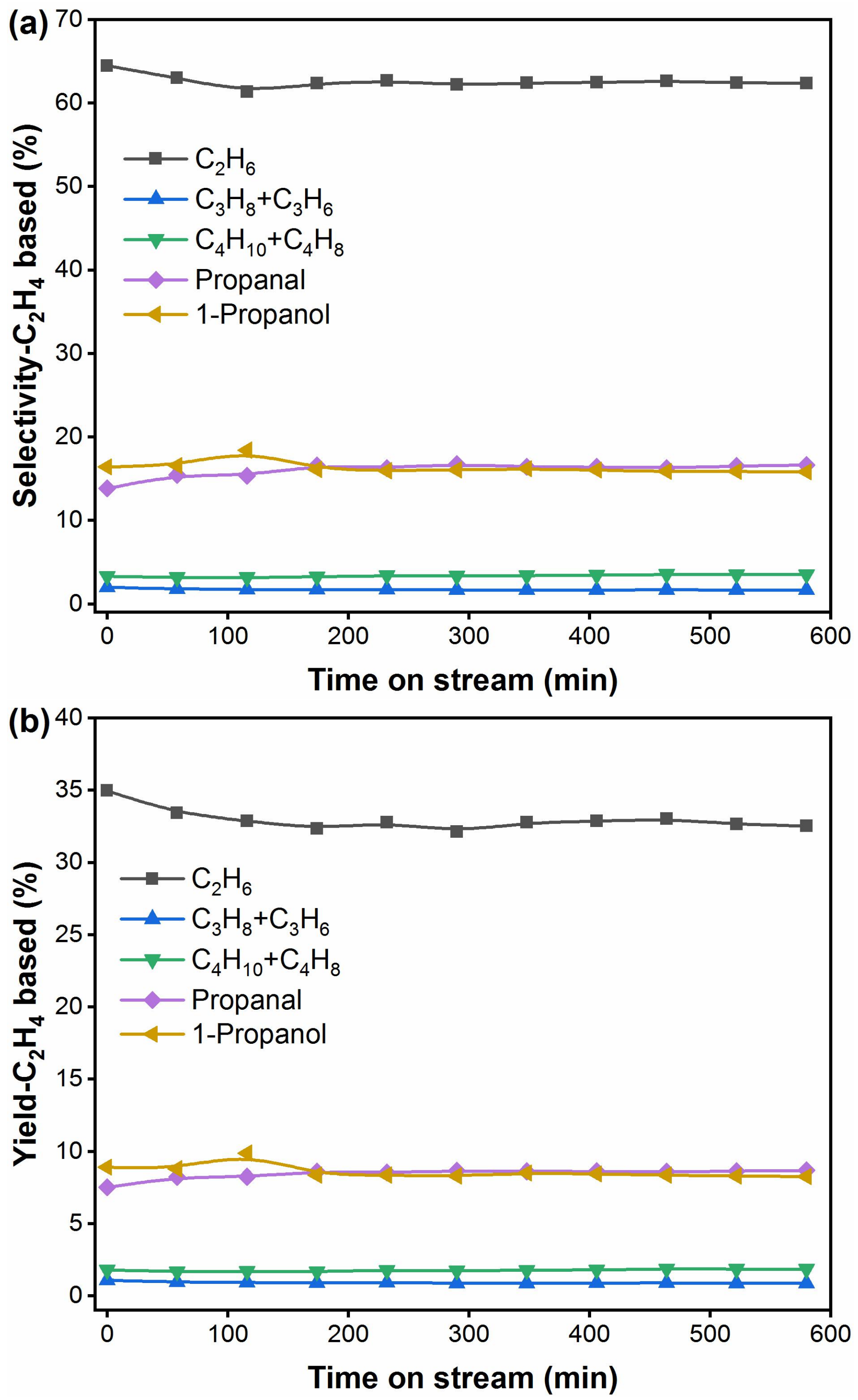

Supplementary Figure 5. Catalytic performance of the Rh_1Co_3/MCM-41 catalyst. **(a)**, **(b)** Selectivity and yield of the carbon containing products. Reaction conditions: 200 ℃, $C_2H_4/CO/H_2/Ar$=3/3/3/3 ml/min, catalyst mass=200 mg, 60-80 mesh, diluted with 100 mg of acid-purified quartz particles (60-80 mesh), atmospheric pressure.

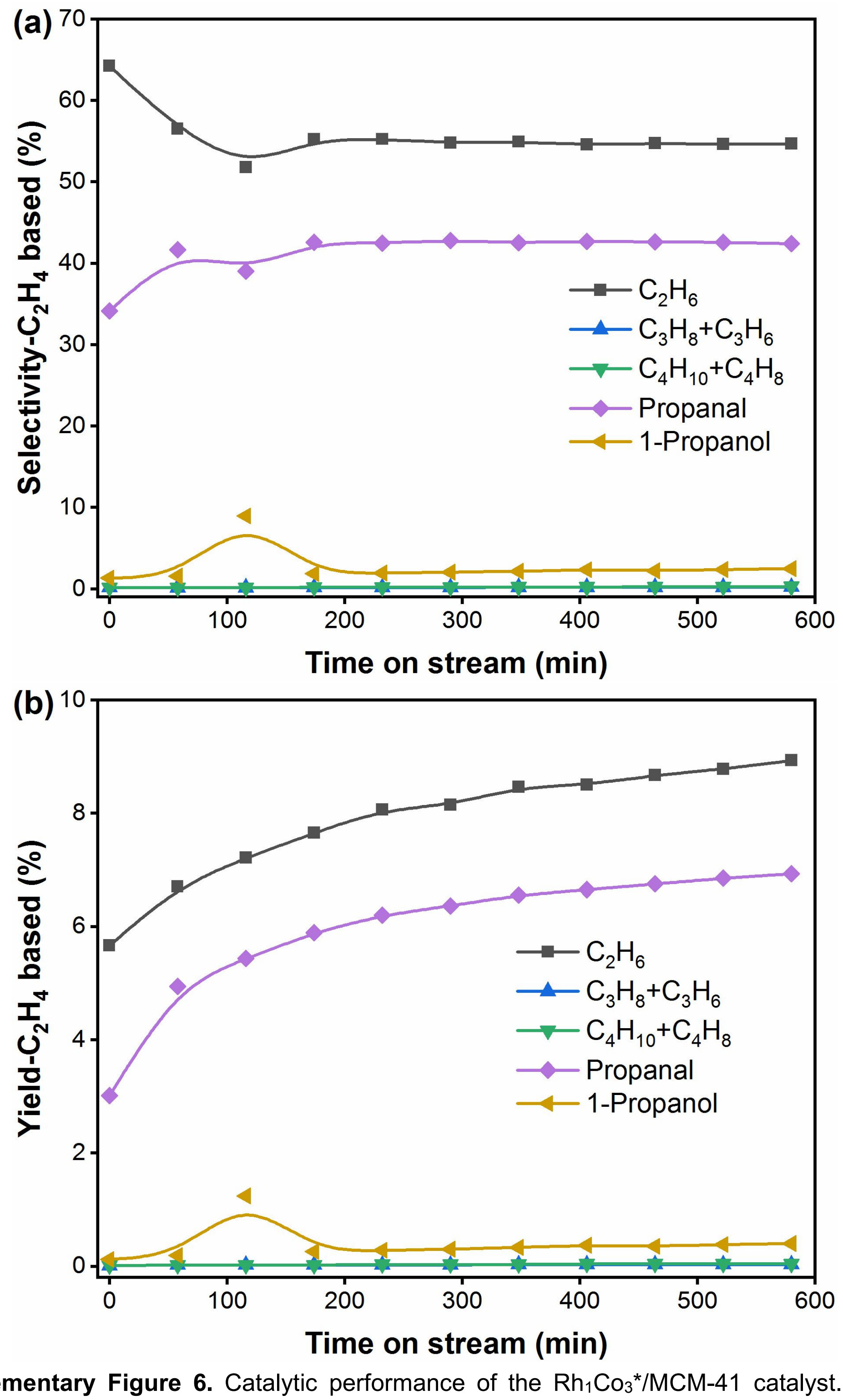

Supplementary Figure 6. Catalytic performance of the Rh_1Co_3*/MCM-41 catalyst. **(a)**, **(b)** Selectivity and yield of the carbon containing products. Reaction conditions: 180 ℃, C_2H_4/CO/H_2/Ar=3/3/3/3 ml/min, catalyst mass=200 mg, 60-80 mesh, diluted with 100 mg of acid-purified quartz particles (60-80 mesh), atmospheric pressure. Note: Rh_1Co_3*/MCM-41 indicates that the 2nd reactor was maintained at 180℃ and loaded with the Rh_1Co_3/MCM-41 catalyst.

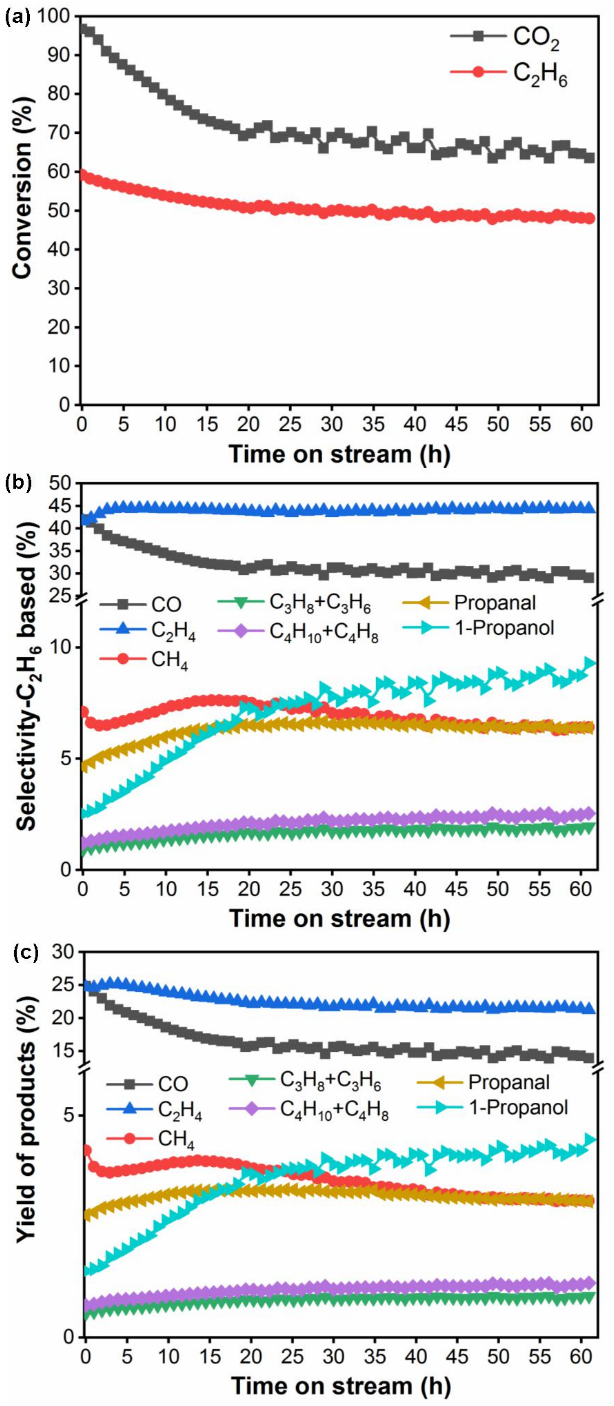

Supplementary Figure 7. Long-term evaluation of the catalytic performance of the tandem reactor configuration. **(a)**, **(b)**, **(c)** Conversion, selectivity, and product yield with the tandem reactor configuration of the 1st reactor (Fe_3Ni_1/CeO_2, 500 mg, $C_2H_6/CO_2/Ar$ = 6/3/3 ml/min, 750 ℃) + the 2nd reactor (Rh_1Co_9/MCM-41, 300 mg, 180 ℃). Note: The Rh_1Co_9/MCM-41 catalyst was synthesized with the same procedure as described in Methods; compared with the best Fe_3Ni_1/CeO_2 (750℃) + Rh_1Co_3/MCM-41 (200 ℃) combination, the selectivity to C3 oxygenates was increased from 4.4% to 14.9% (average between 57-61 h), and the corresponding yields were increased from 1.3% to 7.2% (average between 57-61 h).

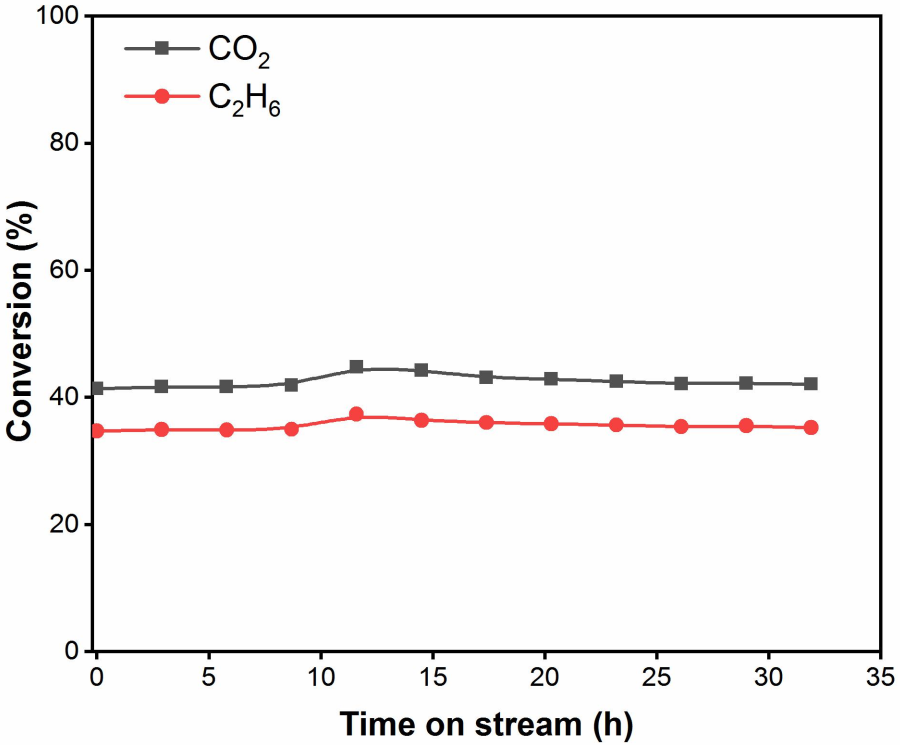

Supplementary Figure 8. Stability test of the spent Fe_3Ni_1/CeO_2 catalyst at 750 ℃. Reaction conditions: $C_2H_6/CO_2/Ar$=6/3/3 ml/min, 300 mg, 60-80 mesh, diluted with 100 mg of acid-purified quartz particles (60-80 mesh), atmospheric pressure. Note: the spent Fe_3Ni_1/CeO_2 catalyst used here was the one that went through the reaction from 600 ℃ to 850 ℃ as shown in Supplementary Table 5; data points were collected every 173 min.

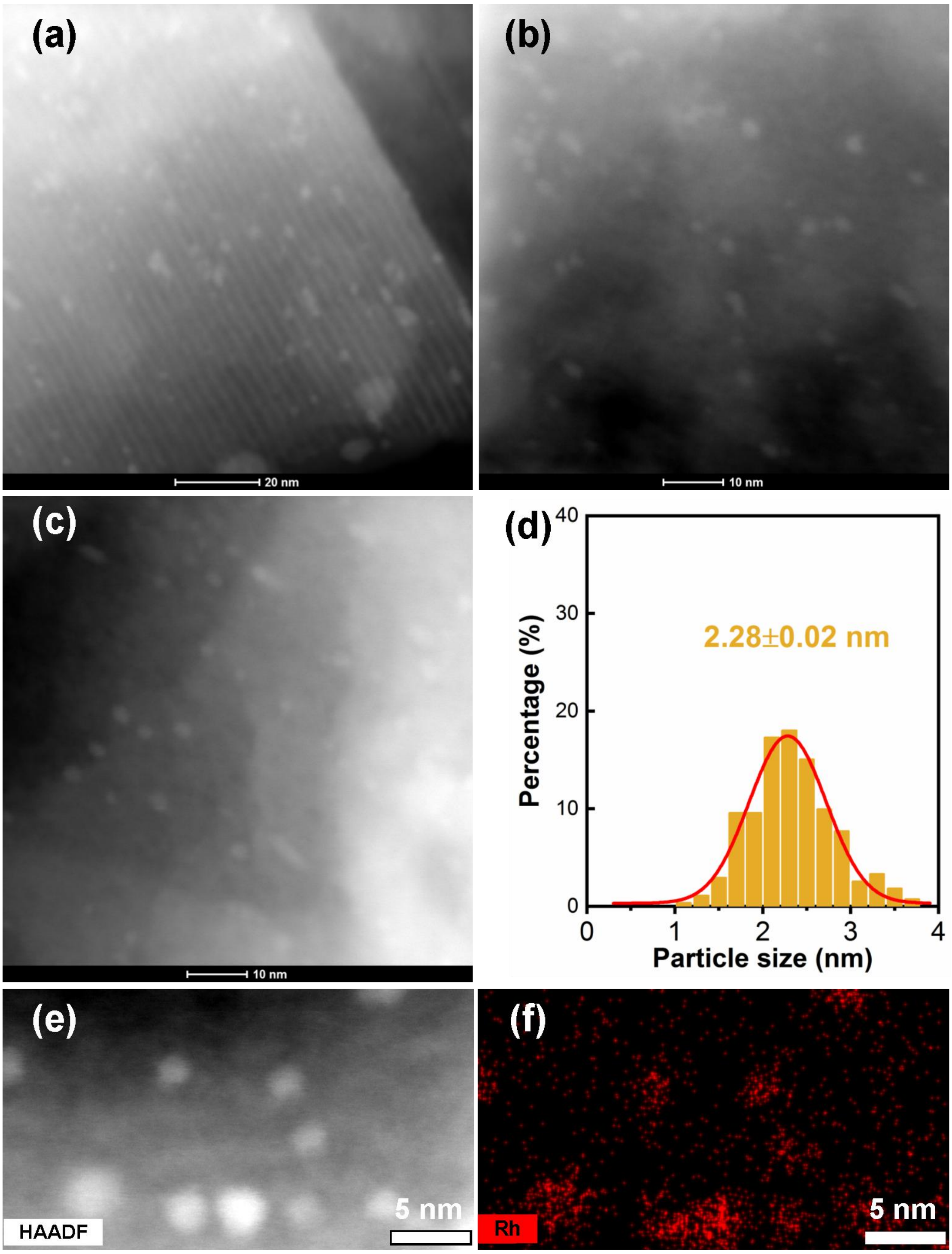

Supplementary Figure 9. Electron microscopy imaging of the spent Rh/MCM-41 catalyst after exposed to hydroformylation reaction stream for 10 h. **(a)**, **(b)**, **(c)**, **(e)** High-angle annular dark-field (HAADF)-scanning transmission electron microscopy (STEM) images. **(d)** Metal cluster/particle distribution. **(f)** Rh EDS mapping.

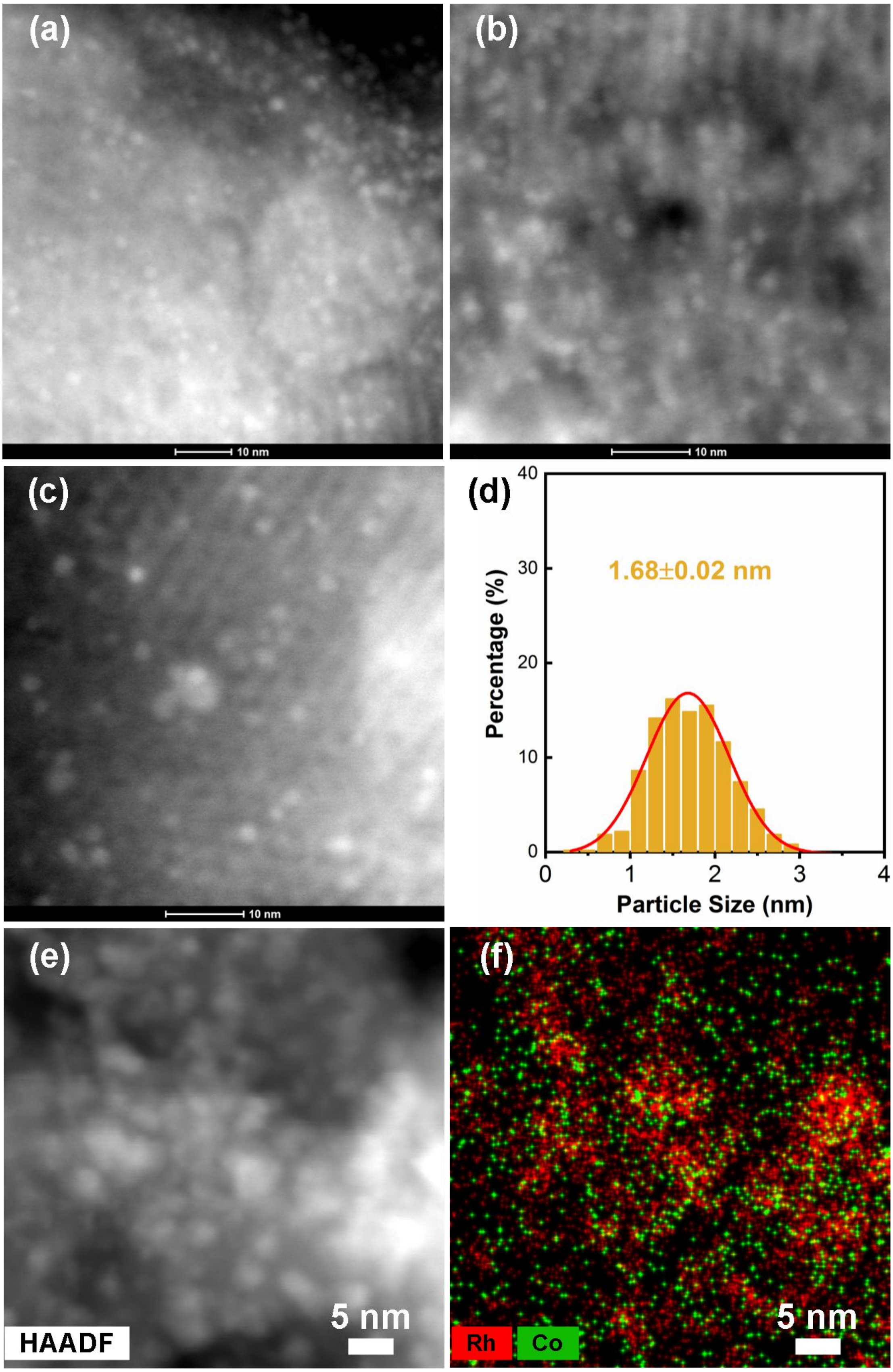

Supplementary Figure 10. Electron microscopy imaging of the spent Rh_1Co_1/MCM-41 catalyst after exposed to hydroformylation reaction stream for 10 h. **(a)**, **(b)**, **(c)**, **(e)** HAADF-STEM images. **(d)** Metal cluster/particle distribution. **(f)** Rh and Co EDS mappings.

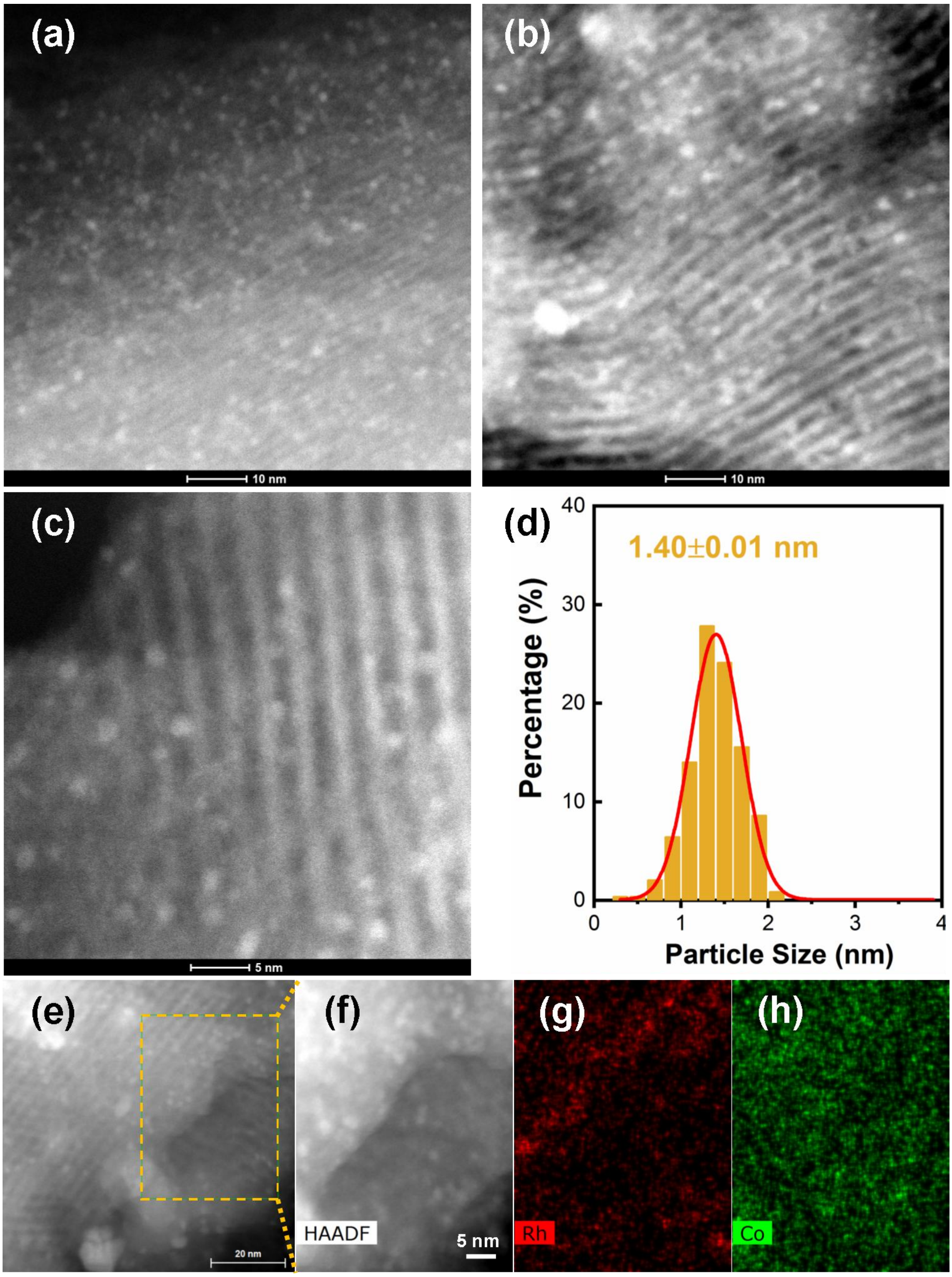

Supplementary Figure 11. Electron microscopy imaging of the spent Rh_1Co_3/MCM-41 catalyst after exposed to hydroformylation reaction stream for 10 h. **(a)**, **(b)**, **(c)**, **(e)**, **(f)** HAADF-STEM images. **(d)** Metal cluster/particle distribution. **(g)**, **(h)** Rh and Co EDS mappings.

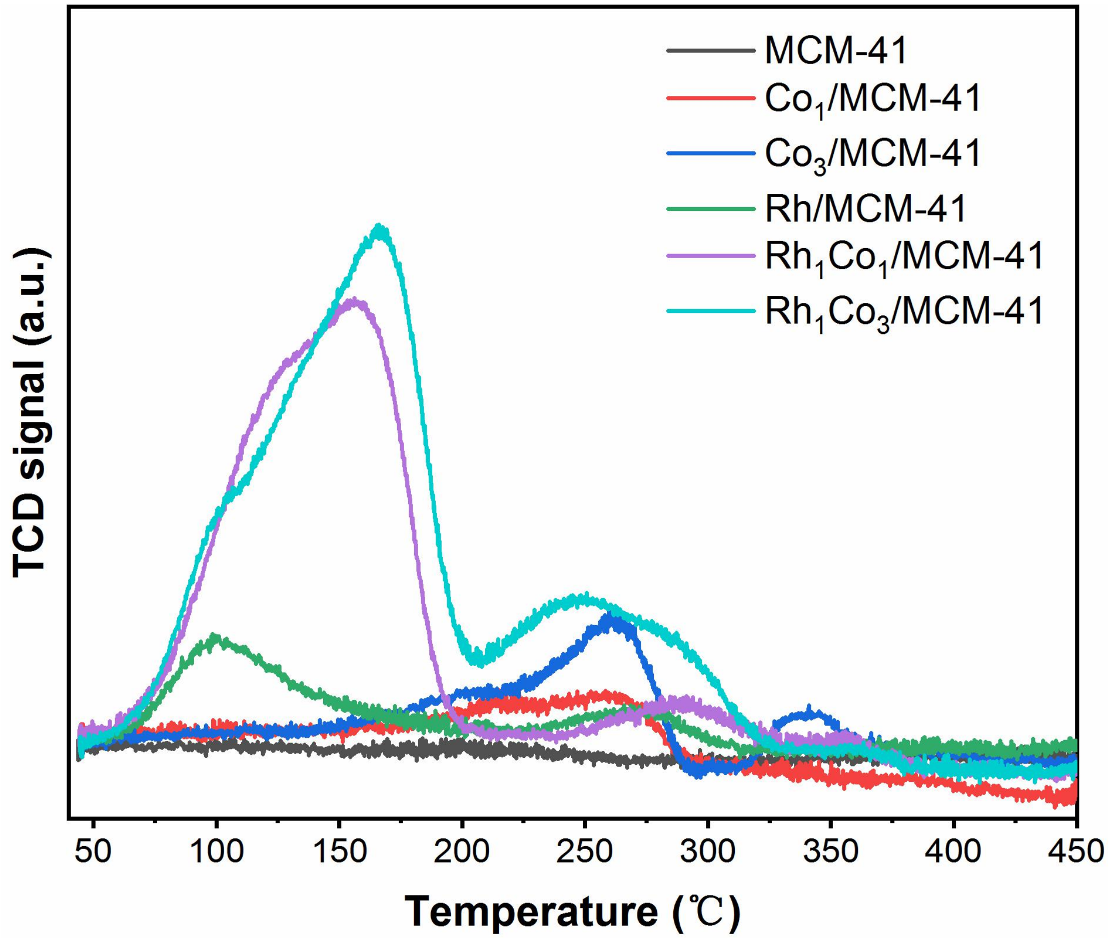

Supplementary Figure 12. Temperature-programmed H_2-reduction profiles of the fresh MCM-41 supported catalysts. Samples: MCM-41, Co_1/MCM-41, Co_3/MCM-41, Rh/MCM-41, Rh_1Co_1-41, and Rh_1Co_3/MCM-41 catalysts.

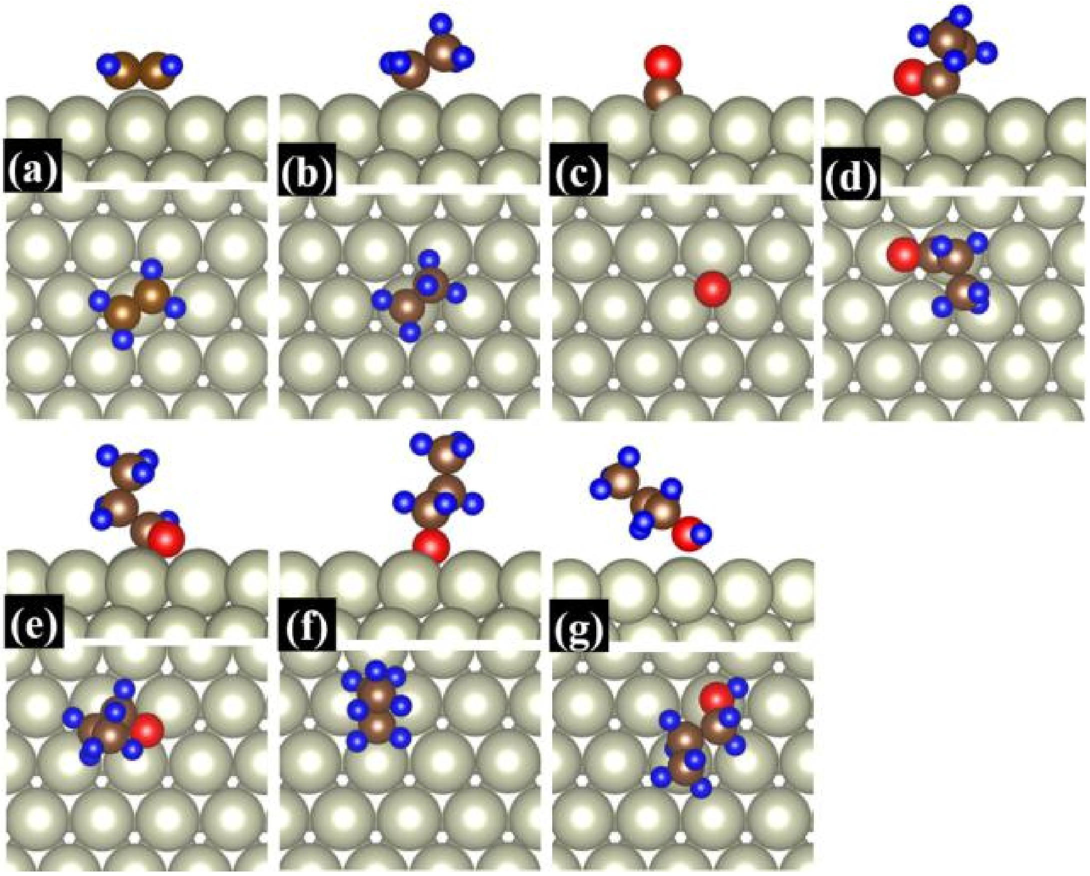

Supplementary Figure 13. DFT optimized geometries of intermediate species on Rh(111). **(a)** *CH_2CH_2. **(b)** *CH_3CH_2. **(c)** *CO. **(d)** *CH_3CH_2CO. **(e)** *CH_3CH_2CHO. **(f)** *$CH_3CH_2CH_2O$. **(g)** *$CH_3CH_2CH_2OH$. Colors: Rh—light green, C—brown, O—red, and H—blue.

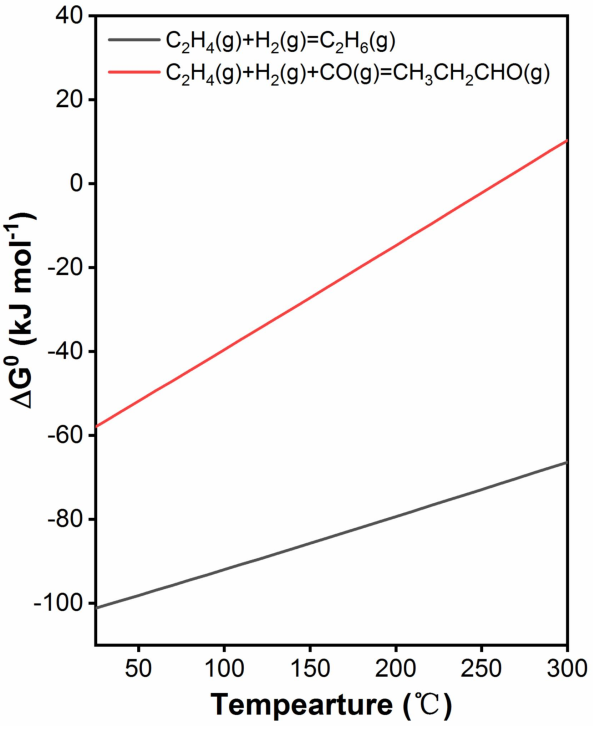

Supplementary Figure 14. Thermodynamic analysis of hydroformylation and hydrogenation reactions of ethylene as a function of temperature.

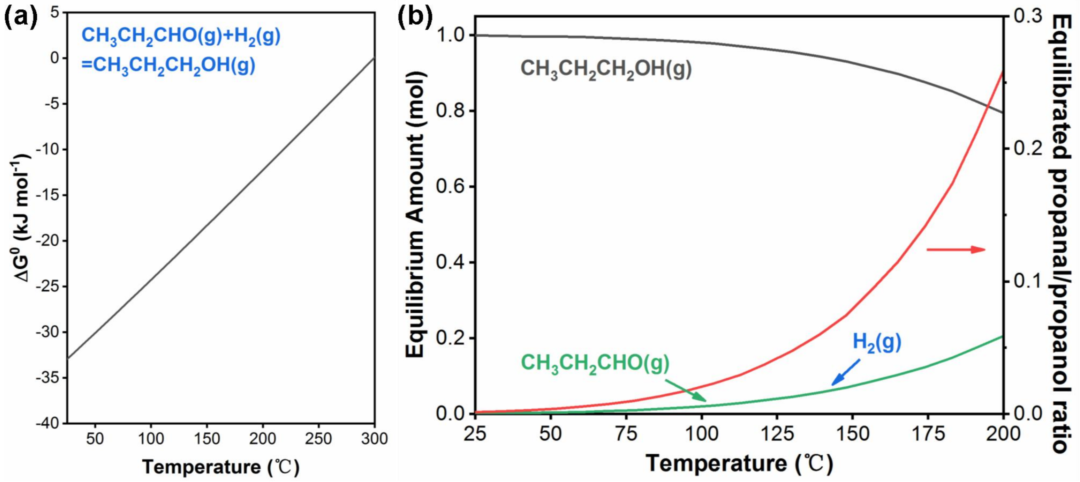

Supplementary Figure 15. Thermodynamic calculations for the hydrogenation of propanal to 1-propanol. **(a)** Standard Gibbs free energy change (ΔG^0). **(b)** Equilibrium amount of species.

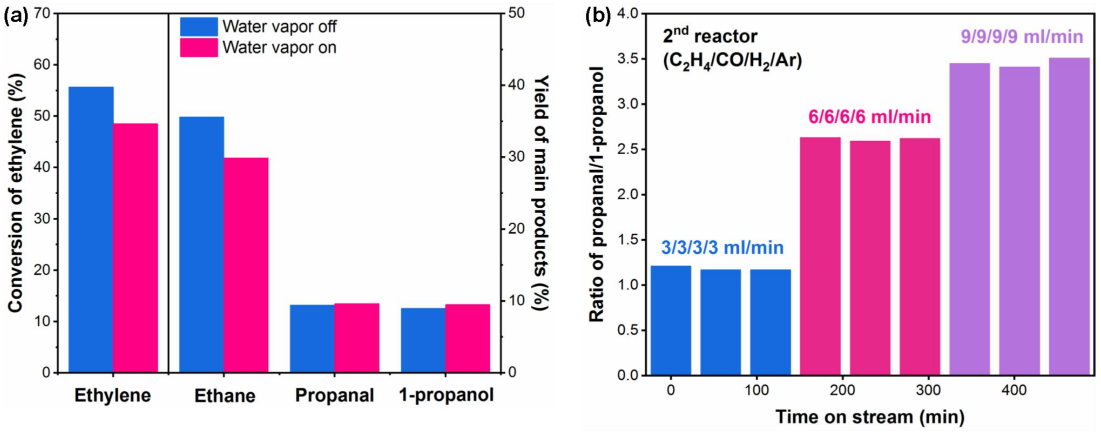

Supplementary Figure 16. Effect of water vapor and residence time on the catalytic performance of the Rh_1Co_3/MCM-41 catalyst. **(a)** Conversion of ethylene and yield of main products. **(b)** Propanal/1-propanol ratio. Reaction conditions: 200 ℃, C_2H_4/CO/H_2/Ar=3/3/3/3 ml/min, catalyst mass=200 mg, 60-80 mesh, diluted with 100 mg of acid-purified quartz particles (60-80 mesh), atmospheric pressure; water vapor was fed into the reactor by passing all the reactants through a bubbler filled with DI water.

Supplementary Table 1 Sample information (metal loading amount, atomic ratio and CO uptake values) of different catalysts.

Catalysts	Metal	Metal loading amount (wt%)	Atomic ratio	CO uptake ($\mu mol/g_{cat}$)
Fe_3Ni_1/CeO_2[a]	Fe[b]	1.43	3:1	-
	Ni[c]	0.50		
Rh/MCM-41[f]	Rh[d]	1.00	1:0	57.6
Rh_1Co_1/MCM-41[f]	Rh[d]	1.00	1:1	78.9
	Co[e]	0.57		
Rh_1Co_3/MCM-41[f]	Rh[d]	1.00	1:3	108.1
	Co[e]	1.72		
Co_3/MCM-41[f]	Co[e]	1.72	0:3	Negligible

[a]CeO_2, 35-45 m^2/g, cubic, Sigma-Aldrich

[b]$Fe(NO_3)_3{\cdot}9H_2O$, 99.999% trace metals basis, Sigma-Aldrich

[c]$Ni(NO_3)_2{\cdot}6H_2O$, 99.999% trace metals basis, Sigma-Aldrich

[d]$Rh(H_2O)(OH)_{3-y}(NO_3)_y$ y=2-3, ~36% rhodium (Rh) basis, Sigma-Aldrich

[e]$Co(NO_3)_2{\cdot}6H_2O$, 99.999% trace metals basis, Sigma-Aldrich

[f]MCM-41, hexagonal, 0.98 cm^3/g pore volume, 2.1-2.7 nm pore size, ~1000 m^2/g, Sigma-Aldrich

Supplementary Table 2 Catalytic performance of MCM-41 supported catalysts within the 2nd reactor at 200 ℃ (C_2H_4/CO/H_2/Ar=3/3/3/3 ml/min, catalyst mass=200 mg, 60-80 mesh, diluted with 100 mg of acid-purified quartz particles (60-80 mesh), atmospheric pressure).

Catalysts	Conversion (%)[a]		TOF (min^{-1})[a]		Selectivity-C_2H_4 based (%)[a]					Yield-C_2H_4 based (%)[a]				
	CO	C_2H_4	CO	C_2H_4	C_2H_6	C_3H_8+C_3H_6	C_4H_{10}+C_4H_8	C_3H_6O	C_3H_8O	C_2H_6	C_3H_8+C_3H_6	C_4H_{10}+C_4H_8	C_3H_6O	C_3H_8O
MCM-41	0.2	0.0	-	-	0.0	4.3	0.0	53.3	42.4	0.0	0.0	0.0	0.0	0.0
Rh/MCM-41	2.0	24.5	0.2	2.6	88.3	0.4	1.2	9.9	0.2	21.6	0.1	0.3	2.4	0.0
Rh_1Co_1/MCM-41	9.8	41.7	0.8	3.5	67.2	0.5	0.6	23.8	7.9	28.0	0.2	0.3	9.9	3.3
Rh_1Co_3/MCM-41	13.1	52.4	0.8	3.2	62.5	1.7	3.5	16.5	15.8	32.8	0.9	1.8	8.6	8.3
Rh_1Co_3*/MCM-41	5.3	16.1	0.3	1.0	54.7	0.2	0.2	42.5	2.4	8.8	0.0	0.0	6.8	0.4
Co_3/MCM-41	0.1	1.2	-	-	97.0	0.3	0.5	1.4	0.8	1.2	0.0	0.0	0.0	0.0

[a]: Quantifications at steady state were calculated based on the data between 8-10 h.

* indicates that the 2nd reactor was maintained at 180 ℃ and loaded with the Rh_1Co_3/MCM-41 catalyst.

Supplementary Table 3 Catalytic performance of blank tube+Rh_1Co_3/MCM-41 within tandem reactors. (C_2H_6/CO_2/Ar=6/3/3 ml/min, blank quartz tube for the 1st reactor (600-800 ℃), 200 mg of Rh_1Co_3/MCM-41 for the 2nd reactor (200 ℃), 60-80 mesh, diluted with 100 mg of acid-purified quartz particles (60-80 mesh), atmospheric pressure).

Temperature (℃)	Conversion (%)		Selectivity-C_2H_6 based (%)							Yield-C_2H_6 based (%)						
	CO_2	C_2H_6	CO	CH_4	C_2H_4	C_3H_8+C_3H_6	C_4H_{10}+C_4H_8	C_3H_6O	C_3H_8O	CO	CH_4	C_2H_4	C_3H_8+C_3H_6	C_4H_{10}+C_4H_8	C_3H_6O	C_3H_8O
600	0.3	0.4	10.8	8.3	41.8	1.4	0.0	0.3	37.4	0.0	0.0	0.2	0.0	0.0	0.0	0.1
650	0.1	0.5	4.4	2.0	87.7	2.7	2.6	0.3	0.3	0.0	0.0	0.4	0.0	0.0	0.0	0.0
700	0.2	2.6	0.8	2.9	85.3	3.2	7.2	0.1	0.5	0.0	0.1	2.2	0.1	0.2	0.0	0.0
750	1.1	9.1	0.9	6.8	73.2	5.3	12.9	0.3	0.6	0.1	0.6	6.7	0.5	1.2	0.0	0.1
800	2.0	18.8	0.0	19.3	46.5	9.7	24.0	0.3	0.2	0.0	3.6	8.7	1.8	4.5	0.1	0.0

Supplementary Table 4 Catalytic performance of quartz+Rh_1Co_3/MCM-41 within tandem reactors. (C_2H_6/CO_2/Ar=6/3/3 ml/min, 300 mg of quartz particles for the 1st reactor (600-800 ℃), 200 mg of Rh_1Co_3/MCM-41 for the 2nd reactor (200 ℃), 60-80 mesh, diluted with 100 mg of acid-purified quartz particles (60-80 mesh), atmospheric pressure).

Temperature (℃)	Conversion (%)		Selectivity-C_2H_6 based (%)							Yield-C_2H_6 based (%)					
	CO_2	C_2H_6	CO	CH_4	C_2H_4	$C_3H_8+C_3H_6$	$C_4H_{10}+C_4H_8$	C_3H_6O	C_3H_8O	CO	CH_4	C_2H_4	$C_3H_8+C_3H_6$	$C_4H_{10}+C_4H_8$	C_3H_6O	C_3H_8O
600	0.0	0.1	2.4	1.2	59.2	0.0	0.0	0.0	37.2	0.0	0.0	0.1	0.0	0.0	0.0	0.0
650	0.1	0.7	4.6	1.1	92.0	0.6	0.4	0.0	1.3	0.0	0.0	0.6	0.0	0.0	0.0	0.0
700	0.6	4.8	2.1	1.5	92.2	1.2	2.5	0.1	0.4	0.1	0.1	4.4	0.1	0.1	0.0	0.0
750	0.8	11.5	0.1	5.7	79.1	4.6	9.5	0.1	0.9	0.0	0.7	9.1	0.5	1.1	0.0	0.1
800	1.8	16.7	0.0	22.6	41.3	11.3	24.6	0.0	0.2	0.0	3.8	6.9	1.9	4.1	0.0	0.0

Supplementary Table 5 Catalytic performance of Fe_3Ni_1/CeO_2 within the 1st reactor. ($C_2H_6/CO_2/Ar$=6/3/3 ml/min, 300 mg of Fe_3Ni_1/CeO_2 for the 1st reactor (600-850 ℃), 60-80 mesh, diluted with 100 mg of acid-purified quartz particles (60-80 mesh), atmospheric pressure).

Temperature (℃)	Conversion (%)		Selectivity-C_2H_6 based (%)							Yield-C_2H_6 based (%)						
	CO_2	C_2H_6	CO	CH_4	C_2H_4	C_3H_8+C_3H_6	C_4H_{10}+C_4H_8	C_3H_6O	C_3H_8O	CO	CH_4	C_2H_4	C_3H_8+C_3H_6	C_4H_{10}+C_4H_8	C_3H_6O	C_3H_8O
600	22.6	7.1	48.4	1.0	50.6	0.0	0.0	0.0	0.0	3.4	0.1	3.6	0.0	0.0	0.0	0.0
650	32.9	12.4	42.3	1.4	56.3	0.0	0.0	0.0	0.0	5.2	0.2	7.0	0.0	0.0	0.0	0.0
700	38.0	18.2	32.5	1.3	65.6	0.1	0.5	0.0	0.0	5.9	0.2	11.9	0.0	0.1	0.0	0.0
750	44.2	36.1	17.8	1.9	78.3	0.6	1.4	0.0	0.0	6.4	0.7	28.3	0.2	0.5	0.0	0.0
800	88.7	67.3	30.0	4.1	63.5	0.8	1.6	0.0	0.0	20.2	2.8	42.7	0.5	1.1	0.0	0.0
850	73.0	87.6	21.0	12.1	63.4	1.0	2.5	0.0	0.0	18.4	10.6	55.5	0.9	2.2	0.0	0.0

Supplementary Table 6 Catalytic performance of Fe_3Ni_1/CeO_2+Rh/MCM-41 within tandem reactors. (C_2H_6/CO_2/Ar=6/3/3 ml/min, 300 mg of Fe_3Ni_1/CeO_2 for the 1st reactor (600-800 ℃), 200 mg of Rh/MCM-41 for the 2nd reactor (200 ℃), 60-80 mesh, diluted with 100 mg of acid-purified quartz particles (60-80 mesh), atmospheric pressure).

Temperature (℃)	Conversion (%)		Selectivity-C_2H_6 based (%)							Yield-C_2H_6 based (%)					
	CO_2	C_2H_6	CO	CH_4	C_2H_4	C_3H_8+C_3H_6	C_4H_{10}+C_4H_8	C_3H_6O	C_3H_8O	CO	CH_4	C_2H_4	C_3H_8+C_3H_6	C_4H_{10}+C_4H_8	C_3H_6O	C_3H_8O
600	19.4	6.3	54.3	1.2	43.0	0.3	0.5	0.4	0.3	3.4	0.1	2.7	0.0	0.0	0.0	0.0
650	32.1	10.9	45.3	1.5	52.1	0.2	0.3	0.4	0.2	4.9	0.2	5.7	0.0	0.0	0.0	0.0
700	36.7	15.8	34.4	1.4	62.7	0.2	0.7	0.5	0.1	5.4	0.2	9.9	0.0	0.1	0.1	0.0
750	42.4	31.6	17.5	2.1	77.0	0.8	1.8	0.7	0.1	5.5	0.7	24.3	0.3	0.6	0.2	0.0
800	90.3	63.8	29.6	4.3	62.8	0.9	1.7	0.6	0.1	18.9	2.7	40.1	0.6	1.1	0.4	0.1

Supplementary Table 7 Catalytic performance of Fe_3Ni_1/CeO_2+Rh_1Co_1/MCM-41 within tandem reactors. (C_2H_6/CO_2/Ar=6/3/3 ml/min, 300 mg of Fe_3Ni_1/CeO_2 for the 1st reactor (600-800 ℃), 200 mg of Rh_1Co_1/MCM-41 for the 2nd reactor (200 ℃), 60-80 mesh, diluted with 100 mg of acid-purified quartz particles (60-80 mesh), atmospheric pressure).

Temperature (℃)	Conversion (%)		Selectivity-C_2H_6 based (%)							Yield-C_2H_6 based (%)					
	CO_2	C_2H_6	CO	CH_4	C_2H_4	C_3H_8+C_3H_6	C_4H_{10}+C_4H_8	C_3H_6O	C_3H_8O	CO	CH_4	C_2H_4	C_3H_8+C_3H_6	C_4H_{10}+C_4H_8	C_3H_6O	C_3H_8O
600	27.9	5.9	76.6	1.0	20.6	0.1	0.1	0.8	0.8	4.5	0.1	1.2	0.0	0.0	0.0	0.0
650	30.0	10.6	43.3	1.6	52.3	0.2	0.3	1.6	0.7	4.6	0.2	5.5	0.0	0.0	0.2	0.1
700	25.5	12.6	24.5	1.6	69.7	0.4	1.2	1.8	0.8	3.1	0.2	8.8	0.1	0.2	0.2	0.1
750	30.5	27.8	11.1	2.8	77.5	1.4	2.8	2.9	1.5	3.1	0.8	21.5	0.4	0.8	0.8	0.4
800	82.4	60.6	26.2	5.2	57.8	1.2	2.3	5.4	1.9	15.9	3.2	35.0	0.7	1.4	3.3	1.2

Supplementary Table 8 Catalytic performance of Fe_3Ni_1/CeO_2+Rh_1Co_3/MCM-41 within tandem reactors. (C_2H_6/CO_2/Ar=6/3/3 ml/min, 300 mg of Fe_3Ni_1/CeO_2 for the 1st reactor (600-800 ℃), 200 mg of Rh_1Co_3/MCM-41 for the 2nd reactor (200 ℃), 60-80 mesh, diluted with 100 mg of acid-purified quartz particles (60-80 mesh), atmospheric pressure).

Temperature (℃)	Conversion (%)		Selectivity-C_2H_6 based (%)							Yield-C_2H_6 based (%)					
	CO_2	C_2H_6	CO	CH_4	C_2H_4	C_3H_8+C_3H_6	C_4H_{10}+C_4H_8	C_3H_6O	C_3H_8O	CO	CH_4	C_2H_4	C_3H_8+C_3H_6	C_4H_{10}+C_4H_8	C_3H_6O	C_3H_8O
600	26.3	6.7	81.3	0.4	14.9	0.1	0.7	0.7	1.9	5.4	0.0	1.0	0.0	0.0	0.0	0.1
650	33.0	12.0	49.0	1.6	46.5	0.3	0.5	1.3	0.8	5.9	0.2	5.6	0.0	0.1	0.2	0.1
700	33.1	16.4	30.0	1.8	64.0	0.5	1.2	1.7	0.8	4.9	0.3	10.5	0.1	0.2	0.3	0.1
750	35.9	30.5	15.3	2.5	74.3	1.1	2.4	2.6	1.8	4.7	0.8	22.7	0.3	0.7	0.8	0.5
800	71.1	56.7	27.4	5.4	54.9	1.5	2.6	4.7	3.5	15.5	3.1	31.1	0.9	1.5	2.7	2.0

Supplementary Table 9 Catalytic performance of Fe_3Ni_1/CeO_2+Rh_1Co_3*/MCM-41 within tandem reactors. (C_2H_6/CO_2/Ar=6/3/3 ml/min, 300 mg of Fe_3Ni_1/CeO_2 for the 1st reactor (600-800 ℃), 200 mg of Rh_1Co_3/MCM-41 for the 2nd reactor (180 ℃), 60-80 mesh, diluted with 100 mg of acid-purified quartz particles (60-80 mesh), atmospheric pressure).

Temperature (℃)	Conversion (%)		Selectivity-C_2H_6 based (%)							Yield-C_2H_6 based (%)						
	CO_2	C_2H_6	CO	CH_4	C_2H_4	C_3H_8+C_3H_6	C_4H_{10}+C_4H_8	C_3H_6O	C_3H_8O	CO	CH_4	C_2H_4	C_3H_8+C_3H_6	C_4H_{10}+C_4H_8	C_3H_6O	C_3H_8O
600	39.2	8.7	92.6	0.6	6.6	0.0	0.0	0.1	0.1	8.1	0.1	0.6	0.0	0.0	0.0	0.0
650	34.8	12.8	46.8	1.3	50.8	0.1	0.1	0.8	0.1	6.0	0.2	6.5	0.0	0.0	0.1	0.0
700	31.7	17.8	28.4	1.5	67.9	0.2	0.6	1.2	0.2	5.1	0.3	12.1	0.0	0.1	0.2	0.0
750	34.7	35.2	13.3	2.2	79.3	1.0	1.9	2.0	0.3	4.7	0.8	27.9	0.4	0.7	0.7	0.1
800	80.6	67.1	25.9	5.1	64.2	1.0	1.9	1.7	0.2	17.4	3.4	43.1	0.7	1.3	1.1	0.1

Supplementary Table 10 EXAFS fitting results of Rh K-edge and Co K-edge for mono- and bimetallic Rh catalysts under the hydroformylation reaction stream.

Samples	Edge	Shell	C.N.	Bond length (Å)	σ^2	ΔE_0 (eV)	R-factor
Rh/MCM-41	Rh-K	Rh-low Z*	2.2(3)	2.02(1)	0.003	6.37	0.007
		Rh-Rh	7.1(2)	2.68(0)	0.006	1.57	
Rh_1Co_1/MCM-41	Rh-K	Rh-low Z*	3.1(2)	2.03(1)	0.003	6.26	0.013
		Rh-Rh	4.5(4)	2.68(0)	0.007	1.60	
		Rh-Co	0.3(2)	2.60(5)	0.007	1.60	
	Co-K	Co-Rh	0.3(2)	2.60(5)	0.007	-1.27	
		Co-O	5.8(3)	2.08(0)	0.008	-1.27	
		Co···Co(Si)	1.3(7)	3.34(5)	0.008	-1.27	
Rh_1Co_3/MCM-41	Rh-K	Rh-low Z*	3.2(2)	2.03(1)	0.003	6.18	0.010
		Rh-Rh	3.2(4)	2.66(1)	0.007	2.43	
		Rh-Co	1.0(3)	2.58(2)	0.007	2.43	
	Co-K	Co-Rh	0.3(2)	2.58(2)	0.007	-1.99	
		Co-O	4.5(1)	2.06(0)	0.008	-1.99	
		Co···Co(Si)	3.9(4)	3.34(1)	0.008	-1.99	
Co_3/MCM-41	Co-K	Co-O	5.5(3)	1.97(1)	0.012	-6.15	0.006
		Co···Co(Si)	3.2(5)	2.87(1)	0.012	-6.15	

* refers to light elements, *i.e.*, C and O.

Supplementary Table 11 DFT calculated binding energies of intermediates on Rh(111) and $Co_3Rh(111)$ surfaces.

Adsorbate	Binding energies/eV	
	Rh(111)	$Co_3Rh(111)$
$*CH_2CH_2$	-0.98	-0.85
$*CH_3CH_2$	-1.73	-1.54
*CO	-1.99	-1.79
$*CH_3CH_2CO$	-2.50	-2.26
$*CH_3CH_2CHO$	-0.66	-0.76
$*CH_3CH_2CH_2O$	-2.48	-2.93
$*CH_3CH_2CH_2OH$	-0.45	-0.40
